# Actin-driven chromosome clustering facilitates fast and complete chromosome capture in mammalian oocytes

**DOI:** 10.1038/s41556-022-01082-9

**Published:** 2023-02-02

**Authors:** Katarina Harasimov, Julia Uraji, Eike Urs Mönnich, Zuzana Holubcová, Kay Elder, Martyn Blayney, Melina Schuh

**Affiliations:** 1 Max Planck Institute for Multidisciplinary Sciences, Göttingen, Germany; 2 Bourn Hall Clinic, Cambridge, UK; 3 Medical Research Council Laboratory of Molecular Biology, Cambridge, UK; 4 Cluster of Excellence ‘Multiscale Bioimaging: from Molecular Machines to Networks of Excitable Cells’ (MBExC), University of Göttingen, Göttingen, Germany

**Keywords:** Cytoskeleton, Developmental biology, Chromosomes

## Abstract

Accurate chromosome segregation during meiosis is crucial for reproduction. Human and porcine oocytes transiently cluster their chromosomes before the onset of spindle assembly and subsequent chromosome segregation. The mechanism and function of chromosome clustering are unknown. Here we show that chromosome clustering is required to prevent chromosome losses in the long gap phase between nuclear envelope breakdown and the onset of spindle assembly, and to promote the rapid capture of all chromosomes by the acentrosomal spindle. The initial phase of chromosome clustering is driven by a dynamic network of Formin-2- and Spire-nucleated actin cables. The actin cables form in the disassembling nucleus and migrate towards the nuclear centre, moving the chromosomes centripetally by interacting with their arms and kinetochores as they migrate. A cage of stable microtubule loops drives the late stages of chromosome clustering. Together, our data establish a crucial role for chromosome clustering in accurate progression through meiosis.

## Abstract (web summary)

Harasimov et al. show that, in human and porcine oocytes, actin cables and microtubule loops move chromosomes into a cluster before spindle assembly to ensure fast and complete chromosome capture in meiosis.

## Main

Human eggs frequently carry an incorrect number of chromosomes^1^, a condition referred to as aneuploidy. Most aneuploid eggs cannot develop to term upon fertilization, making high levels of aneuploidy in eggs a leading cause of pregnancy loss and infertility in women^1–3^. Aneuploidy in eggs arises from chromosome segregation errors during oocyte meiosis^1,4^. Despite its fundamental importance for fertility and development, our understanding of human oocyte meiosis is limited.

Chromosomes are segregated by a microtubule spindle, which in human oocytes assembles in the absence of centrosomes^5,6^. Defects during spindle assembly were linked to high aneuploidy levels in human eggs^5,7^. However, the mechanisms that facilitate spindle assembly in human oocytes remain largely unclear.

The nucleation of spindle microtubules after nuclear envelope breakdown (NEBD) is markedly delayed in human oocytes compared with mitotic cells^8^ or mouse oocytes^9^. Interestingly, chromosomes in human oocytes undergo rapid movements after NEBD and before the onset of spindle assembly^5^. Upon NEBD, the chromosomes migrate centripetally towards each other, forming a compact chromosome cluster^5^. The chromosome cluster remains intact until a microtubule aster forms within the cluster’s centre^5^. The aster grows and transforms into a bipolar spindle^5^. The physiological relevance of chromosome clustering in human oocytes and its underlying mechanism are unknown.

Chromosomes in starfish and *Xenopus* oocytes are also transported before spindle assembly. The nuclei of these oocytes are over two times larger than those of human oocytes and their chromosomes must be moved to bring them within reaching distance of spindle microtubules^10,11^. In starfish oocytes, each chromosome becomes enclosed by a dense layer of actin^12^, and then linked to a filamentous actin (F-actin) network^13^ that contracts towards the centrosomes^14^. Once the chromosomes are within reaching distance^14^, the centrosomes rapidly assemble the meiotic spindle^12,13^. In *Xenopus* oocytes, the chromosomes are transported by a transient microtubule structure that assembles from an acentriolar microtubule organizing centre on the cytoplasmic side of the nuclear envelope^15^. The transient microtubule array associates with condensed chromosomes, rapidly migrates towards the cell cortex and transforms into the first mitotic spindle^15^. Notably, starfish and *Xenopus* oocytes do not form chromosome clusters during meiosis I. Whether the mechanisms of chromosome movement in starfish and *Xenopus* oocytes are relevant to the chromosome clustering observed in human oocytes is unclear.

The actin cytoskeleton plays a role in positioning chromosomes during mitosis of human cells^16^. The actomyosin network in mitotic cells forms in the cytoplasm around the nucleus during prophase and contracts upon entry into mitosis, thereby reducing the nuclear volume and consequently the volume occupied by the chromosomes^16^. Similar to starfish and *Xenopus* oocytes, this facilitates the capture of chromosomes by microtubules upon NEBD^16^.

In this Article, we investigated the mechanism and function of chromosome clustering in human and porcine oocytes. We used the porcine model system, as oocytes and early embryos of pigs closely resemble those of humans^7,17–22^. Additionally, pigs resemble humans genetically, physiologically and anatomically^23–25^. Although mouse oocytes are a commonly used mammalian model, unlike human oocytes, they rapidly assemble a spindle after NEBD^26^ and do not form a chromosome cluster^9,26^.

We report that chromosome clustering in human and porcine oocytes is predominantly driven by actin cables that interact with the chromosomes’ kinetochores and arms. A cage of microtubule loops forms around the chromosomes in the late stages of NEBD and transports chromosomes that have not yet been clustered by the actin cables. Chromosome clustering promotes rapid and complete capture of all chromosomes by the assembling spindle. Chromosome clustering is therefore an essential stage of porcine and human oocyte development.

## Results

### Human and porcine oocytes cluster their chromosomes

**Fig. 1:**
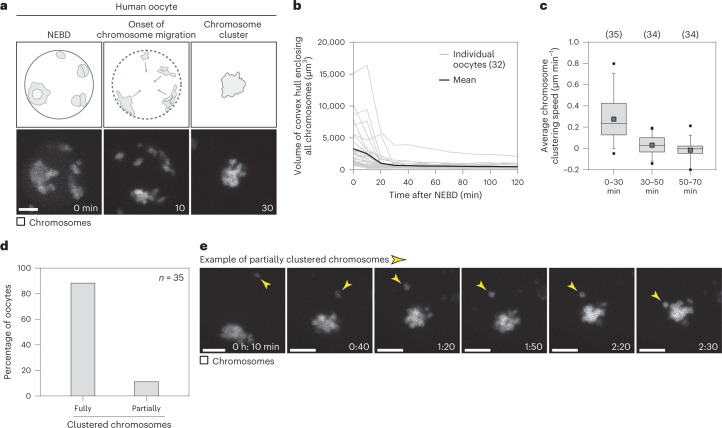
Human oocytes cluster their chromosomes upon NEBD. **a**, Chromosome clustering in human oocytes with dispersed chromosomes at NEBD. Top row: scheme illustrating chromosome configuration (chromatin in grey, nuclear envelope in black). Bottom row: live human oocyte expressing histone 2B–monomeric red fluorescent protein 1 (H2B-mRFP1) (chromosomes) and enhanced green fluorescent protein–microtubule-associated protein 4 (EGFP-MAP4) (not shown). *Z*-projections, six sections every 5 µm. **b**, Volume of convex hull enclosing all chromosomes reconstructed from videos of human oocytes. **c**, Box plot showing average chromosome clustering speed at indicated time intervals. Box plot shows median (horizontal black line), mean (small black squares), 25th and 75th percentiles (boxes), 5th and 95th percentiles (whiskers), and 1st and 99th percentiles (dots). **d**, Bar plot showing percentage of human oocytes with fully or partially clustered chromosomes. **e**, Human oocyte expressing H2B-mRFP1 (chromosomes) and EGFP-MAP4 (not shown). Arrowheads point to a slowly migrating chromosome that does not integrate into the cluster. *Z*-projections, five sections every 5 µm. Scale bar, 10 µm. Time, minutes (min) or hours:minutes (h:min) from NEBD. Number of oocytes in brackets (**b** and **c**) or indicated with *n* (**c**). Source numerical data are available in source data. Source data

**Extended Data Fig. 1:**
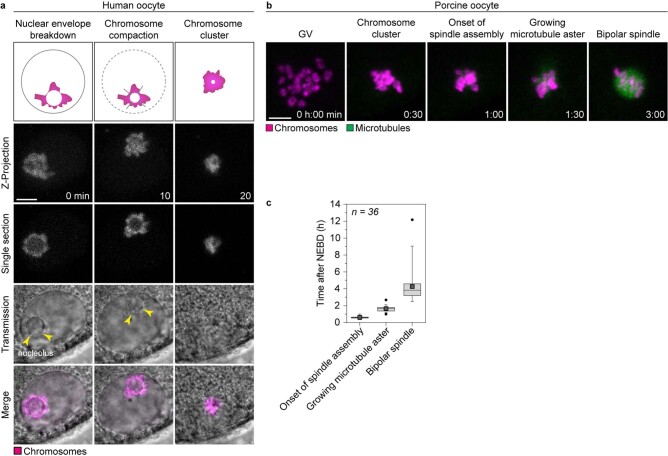
Additional patterns of chromosome clustering in human oocytes. **a**, Chromosome clustering in class of human oocytes with pre-clustered chromosomes at NEBD. Top row: Scheme illustrating chromosome configuration (chromatin in magenta; nuclear envelope in black); Rows bellow: Live human oocyte expressing H2B-mRFP1 (chromosomes) and EGFP-MAP4 (not shown). Yellow arrowheads point to the nucleolus. Z-projection panel, 5 sections every 5 µm. **b**, Porcine oocyte expressing EGFP-MAP4 (microtubules) and H2B-mCherry (chromosomes). Z-projections, 15 sections every 3 µm. **c**, Quantification of timing of meiotic progression from live porcine oocytes as shown in **b**. Box plot shows median (horizontal black line), mean (small black squares), 25th and 75th percentiles (boxes), 5th and 95th percentiles (whiskers) and 1st and 99th percentiles (dots). Scale bar, 10 µm. Time, minutes (min) or hours:minutes (h:min) from NEBD. Number of oocytes indicated with *n*. Data from at least three independent experiments. Source numerical data are available in source data. Source data

We first characterized chromosome clustering in human oocytes and observed two distinct chromatin configurations in the intact nucleus. The chromosomes were either distributed throughout the intact nucleus in multiple clusters before NEBD (43% of oocytes) (Fig. 1a and Supplementary Video 1), or associated with the nucleolus and hence partially clustered before NEBD (57% of oocytes) (Extended Data Fig. 1a and Supplementary Video 1). In both cases, chromosomes moved towards each other rapidly and typically formed a cluster within 30 min after NEBD (Fig. 1b,c and Extended Data Fig. 1a). The clusters were of a similar volume in different oocytes (Fig. 1b). Ultimately, 89% of human oocytes formed a compact chromosome cluster before the onset of spindle formation (Fig. 1a,d,e).

**Fig. 2:**
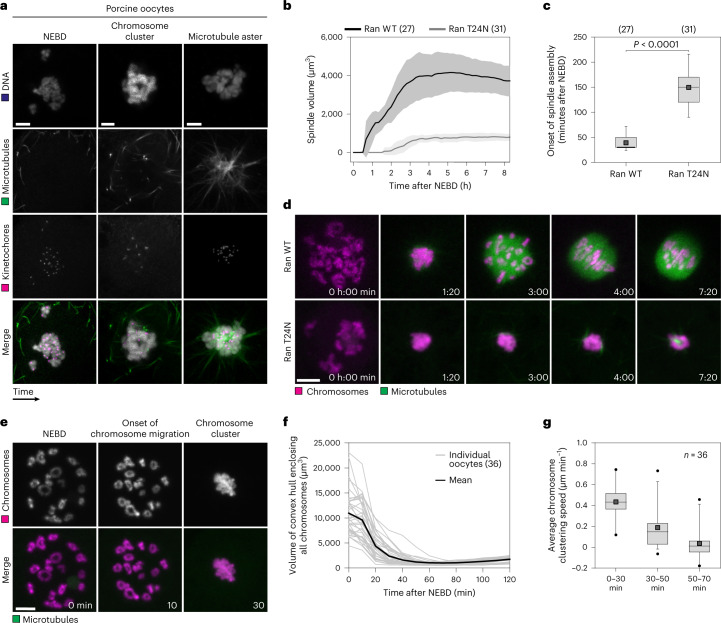
Porcine oocytes cluster their chromosomes upon NEBD. **a**, Immunofluorescence airyscan images of porcine oocytes fixed at different times shortly after NEBD. Green, microtubules (α-tubulin); magenta, kinetochores (ACA); grey, DNA (Hoechst). *Z*-projections, 30 (NEBD) and 18 sections (microtubule aster) every 0.19 µm. **b**, Quantification of the spindle volume in porcine oocytes as shown in **d** based on EGFP-MAP4 signal. Data are presented as mean ± standard deviation (s.d.). **c**, Onset of microtubule nucleation in live porcine oocytes as shown in **d**. *P* < 0.0001 (two-tailed Mann–Whitney test). **d**, Live porcine oocytes expressing H2B-mCherry (chromosomes), EGFP-MAP4 (microtubules) and (wildtype Ran) Ran WT-monomeric near-infrared fluorescent protein 670 (miRFP670) (upper panel) or Ran T24N-miRFP670 (lower panel). *Z*-projections, 15 sections every 3 µm. **e**, Chromosome clustering in live porcine oocyte expressing EGFP-MAP4 (microtubules) and H2B–mCherry (chromosomes). *Z*-projections, 15 sections every 3 µm. **f**, Volume of convex hull enclosing all chromosomes reconstructed from videos of porcine oocytes as shown in **e**. **g**, Box plot showing average chromosome clustering speed at indicated time intervals. Scale bars, 10 µm (**d** and **e**) and 5 µm (**a**). Time, hours:minutes (h:min) or minutes (min) from NEBD. Number of oocytes in brackets or indicated with *n*. Data from at least three independent experiments. Box plots show median (horizontal black line), mean (small black squares), 25th and 75th percentiles (boxes), 5th and 95th percentiles (whiskers) and 1st and 99th percentiles (dots). Source numerical data are available in source data. Source data

Studies in human oocytes are limited by the scarcity of available material. We hence studied meiosis in live porcine oocytes, which closely resemble human oocytes^7,17–22^. Spindle organization is similar in porcine and human oocytes^7^. Moreover, we found that spindle assembly was also similar in porcine and human oocytes. After the onset of NEBD, the chromosomes moved into a cluster (Fig. 2a, Extended Data Fig. 1b and Supplementary Video 2). Subsequently, a microtubule aster appeared within the chromosome cluster (Fig. 2a and Extended Data Fig. 1b). The time between NEBD and microtubule aster formation, however, was only about 1.6 h in porcine oocytes (Extended Data Fig. 1c) compared with ~5 h in human oocytes^5^. Like in human oocytes^5^, spindle assembly in porcine oocytes depended on the small GTPase Ran (Fig. 2b–d and Supplementary Video 3), whose inhibition led to a massive reduction in spindle volume (Fig. 2b) and a delay in the onset of microtubule spindle assembly (Fig. 2c). Thus, the spindle assembly mechanisms in porcine and human oocytes are closely related and involve chromosome clustering.

**Extended Data Fig. 2:**
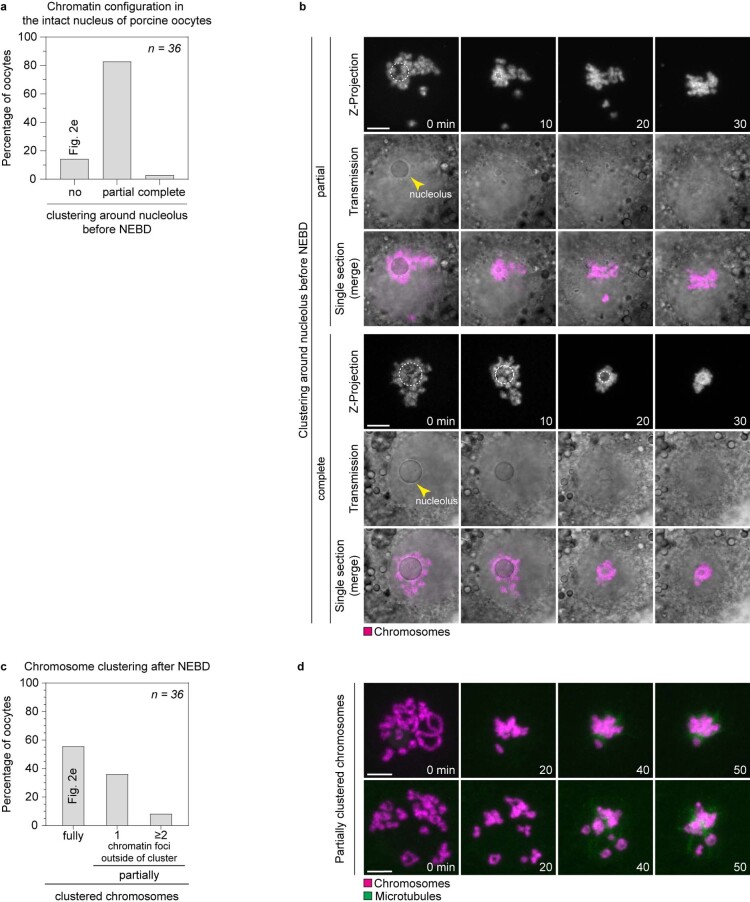
Additional patterns of chromosome clustering in porcine oocytes. **a**, Bar plot showing percentage of porcine oocytes with different levels of chromatin clustering around the nucleolus prior to NEBD. **b**, Porcine oocytes expressing H2B-mCherry (chromosomes) and EGFP-MAP4 (not shown). Examples of the classes of chromatin configurations presented in **a**. Yellow arrowheads point at the nucleolus. Dashed circles demark nucleolus. Z-projection panels, 15 sections every 3 µm. **c**, Bar plot showing percentage of porcine oocytes that fully or partially clustered their chromosomes after NEBD. **d**, Porcine oocytes expressing EGFP-MAP4 (microtubules) and H2B-mCherry (chromosomes). Examples of partial chromatin clustering after NEBD. Z-projections, 15 sections every 3 µm. Scale bar, 10 µm. Time, minutes (min) from NEBD. Number of oocytes indicated with *n*. Data from three independent experiments. Source numerical data are available in source data. Source data

**Extended Data Fig. 3:**
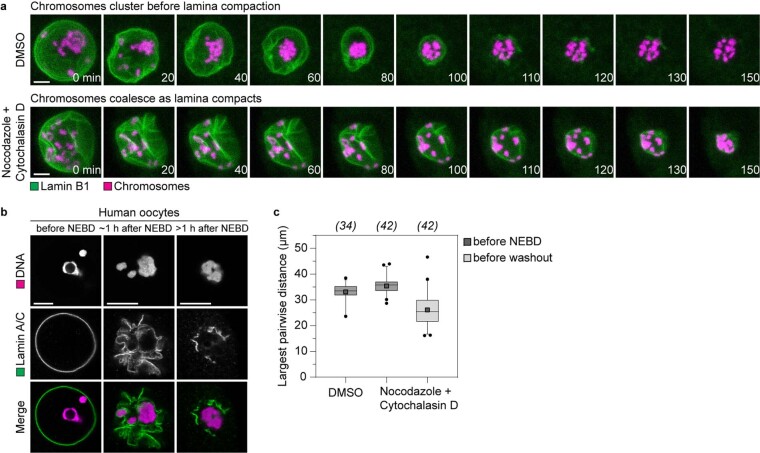
Nuclear lamina compacts together with chromosomes in the absence of microtubules and F-actin. **a**, Live porcine oocytes expressing EGFP-Lamin B1 and H2B-mCherry (chromosomes) treated with DMSO or nocodazole and cytochalasin D. Z-projections, 6 sections every 3 µm. **b**, Immunofluorescence airyscan images of human oocytes before and at different time points after NEBD. Magenta, DNA (Hoechst); green, Lamin A/C; F-actin (not shown); alpha-tubulin (not shown). Representative examples of 53 immunolabeled human oocytes. **c**, Quantification of the largest pairwise distance calculated from the chromosome signal in control or nocodazole and cytochalasin D co-treated porcine oocytes before NEBD and right before drug (nocodazole and cytochalasin D) washout. Box plot shows median (horizontal black line), mean (small black squares), 25th and 75th percentiles (boxes), 5th and 95th percentiles (whiskers) and 1st and 99th percentiles (dots). Scale bar, 10 µm. Time, minutes (min) after NEBD. Number of oocytes in brackets. Data from three independent experiments unless otherwise specified. Source numerical data are available in source data. Source data

We then studied the chromosome clustering phase in porcine oocytes in more detail and found that the chromatin distribution in the nucleus of pig oocytes before NEBD was similar to that in human oocytes (Supplementary Video 2). In most porcine oocytes, the chromosomes were scattered throughout the nucleus before NEBD (Fig. 2e and Extended Data Fig. 2a), although they partially associated with the nucleolus in some oocytes (Extended Data Fig. 3a,b). The chromosomes moved rapidly towards each other during the first ~30 min post-NEBD in porcine oocytes, as in human oocytes, (Fig. 2e–g and Supplementary Video 2), with an average speed of 0.43 µm min^−1^ (Fig. 2g). Subsequently, the chromosomes slowed down, until a cluster had formed (Fig. 2g). Fifty-six per cent of porcine oocytes eventually formed a chromosome cluster that comprised all chromosomes (Fig. 2e and Extended Data Fig. 2c). The remaining 44% formed a large chromosome cluster with one or a few chromatin foci located nearby (Extended Data Fig. 2c,d). The progression and degree of chromosome clustering were thus similar in porcine and human oocytes, although the overall speed of the chromosomes was slightly higher in porcine oocytes, particularly during the second slower phase of chromosome clustering.

### Actin and microtubules drive chromosome clustering

**Fig. 3:**
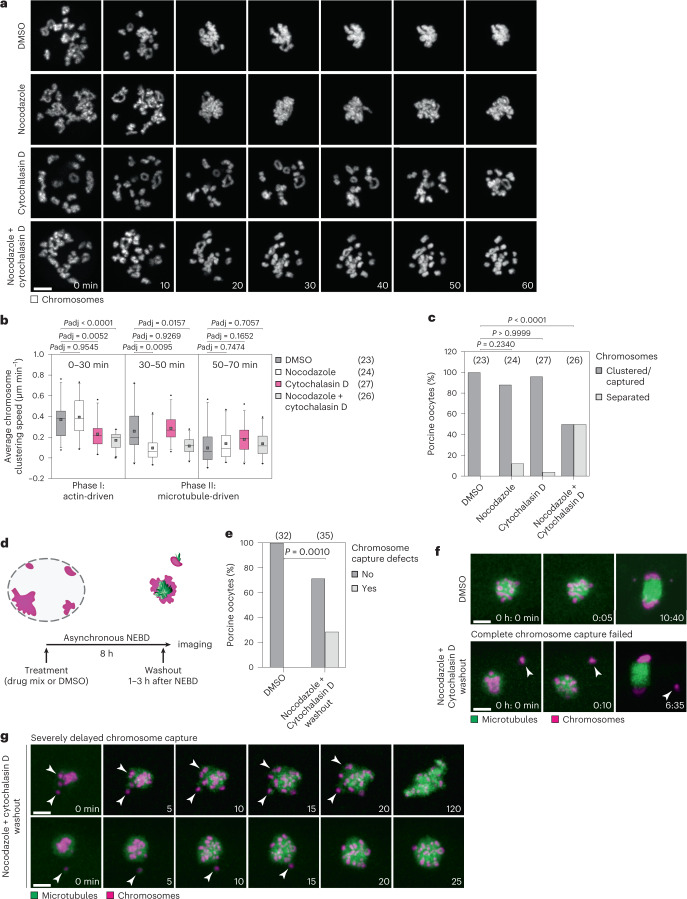
Chromosome clustering is driven by actin and microtubule cytoskeleton and is important for rapid and complete chromosome capture after NEBD. **a**, Porcine oocytes expressing H2B-mCherry (chromosomes) treated with DMSO, nocodazole, cytochalasin D or a mix of nocodazole and cytochalasin D. *Z*-projections, 15 sections every 3 µm. **b**, Quantification of average chromosome clustering speed at indicated time intervals after NEBD in porcine oocytes treated with DMSO, nocodazole, cytochalasin D or combination of nocodazole and cytochalasin D as shown in **a**. Statistical test: Brown–Forsythe and Welch one-way ANOVA tests with Dunnett’s T3 multiple comparisons test. **c**, Bar plot of percentage of porcine oocytes as shown in **a** that cluster or capture chromosomes. Each drug treatment group was compared with the control group using two-sided Fisher’s exact test. **d**, Scheme of the experimental setup for cytochalasin and nocodazole washout experiments (scheme; chromosomes in magenta, microtubules in green, nuclear envelope in grey). **e**, Bar plot showing percentage of cells with chromosome capture defects in porcine oocytes as shown in **f** and **g**. *P* = 0.0010 (two-sided Fisher’s exact test). **f**, Porcine oocytes expressing EGFP-MAP4 (microtubules) and H2B-mCherry (chromosomes). Top: control (*Z*-projection, 6 sections every 3 µm). Bottom: transient treatment around NEBD with cytochalasin D and nocodazole as illustrated in **d** (*Z*-projection, 11 sections every 3 µm). White arrowheads point to a chromosome lost in cytoplasm. **g**, Examples of severely delayed chromosome capture following nocodazole and cytochalasin D washout. *Z*-projection, nine (top) or eight (bottom) sections every 3 µm. Scale bar, 10 µm. Time, minutes (min) from NEBD (**a**) or hours:minutes (h:min)/minutes (min) from imaging onset (**f** and **g**). Data from three independent experiments. Box plots show median (horizontal black line), mean (small black squares), 25th and 75th percentiles (boxes), 5th and 95th percentiles (whiskers) and 1st and 99th percentiles (dots). *P* values indicated above the box plots. Source numerical data are available in source data. Source data

Actin or microtubules move chromosomes into the proximity of the microtubule spindle in starfish^13^ and *Xenopus*^15,27^ oocytes, respectively. To test whether actin and microtubules play a role in chromosome clustering in porcine oocytes, we treated oocytes with either cytochalasin D (actin depolymerization drug) or nocodazole (microtubule depolymerization drug), or with both drugs simultaneously (Fig. 3a), and compared the speed (Fig. 3b) and the efficiency of chromosome clustering (Fig. 3c) with that of control oocytes.

During the first 30 min post-NEBD, the chromosomes moved rapidly in both control oocytes, and oocytes treated with nocodazole (Fig. 3b). However, the speed of clustering was significantly reduced in cells treated with cytochalasin D and with a combination of the drugs (Fig. 3b). Between 30 min and 50 min post-NEBD, the speed of chromosome clustering of oocytes treated with cytochalasin D closely resembled that of control oocytes, whereas in oocytes treated with nocodazole and with the combination of the drugs the speed of clustering was significantly reduced (Fig. 3b). These data suggest that the first phase of chromosome clustering, which lasts between 0 min and 30 min post-NEBD, is driven by the actin cytoskeleton. The second phase, starting from 30 min post-NEBD, is dependent on microtubules.

Next, we asked whether the drug treatments perturbed the efficiency of chromosome clustering. Treatment with either nocodazole or cytochalasin D alone led to a small increase in dispersed or uncaptured chromosomes after NEBD (Fig. 3c). Co-treatment with both drugs had a strong effect and blocked chromosome clustering in half of the oocytes (Fig. 3c). Residual chromosome clustering in oocytes co-depleted for F-actin and microtubules could be driven by the oocytes’ nuclear lamina, which persisted after NEBD for a prolonged time in both porcine (Extended Data Fig. 3a) and human oocytes (Extended Data Fig. 3b), and coalesced together with the chromosomes into a cluster when actin and microtubules were absent in porcine oocytes (Extended Data Fig. 3a and Supplementary Video 4). Thus, actin- and microtubule-dependent chromosome clustering mechanisms act cooperatively to ensure efficient chromosome clustering.

### Clustering promotes capture of chromosomes by microtubules

Next, we investigated the physiological function of chromosome clustering. Pharmacological inhibition of actin and microtubule polymerization led to an increase in the number of oocytes that failed to cluster their chromosomes (Fig. 3c), but the persistent presence of the drugs in this experiment precluded the analysis of effects on subsequent spindle assembly. To overcome this limitation, we transiently treated oocytes with cytochalasin D and nocodazole during the chromosome clustering phase, followed by washout of the drugs (Fig. 3d). At NEBD, chromosomes in both DMSO-treated control oocytes and oocytes co-treated with nocodazole and cytochalasin D were similarly distributed, demonstrating that depolymerization of F-actin and microtubules did not affect chromosome distribution (Extended Data Fig. 3c). Oocytes with dispersed chromosomes were washed into drug-free medium after NEBD, and imaged live as they progressed through meiosis (Fig. 3d).

All chromosomes were captured by the spindle in 100% of control oocytes (Fig. 3e,f). In contrast, 29% of oocytes transiently co-treated with nocodazole and cytochalasin D (Fig. 3e,f) showed defects during chromosome capture; chromosomes either failed to be captured altogether (6% of oocytes), leading to spindles that did not contain the full set of chromosomes (Fig. 3f); or chromosome capture was severely delayed (23% of oocytes) (Fig. 3g), and chromosomes remained outside of the spindle region for prolonged time periods. Thus, chromosome clustering promotes the rapid and complete capture of chromosomes by the assembling spindle.

### Clustering is driven by a dynamic network of actin cables

Having established the importance of chromosome clustering for the formation of the first meiotic spindle, we next investigated the mechanisms that drive chromosome clustering.

Our experiments established that the first phase of chromosome clustering is dependent on F-actin. Additionally, in 57% of live porcine oocytes, chromosome clustering was completed before microtubule nucleation became apparent (Extended Data Fig. 2c). This suggests that chromosome clustering is predominantly driven by F-actin.

**Fig. 4:**
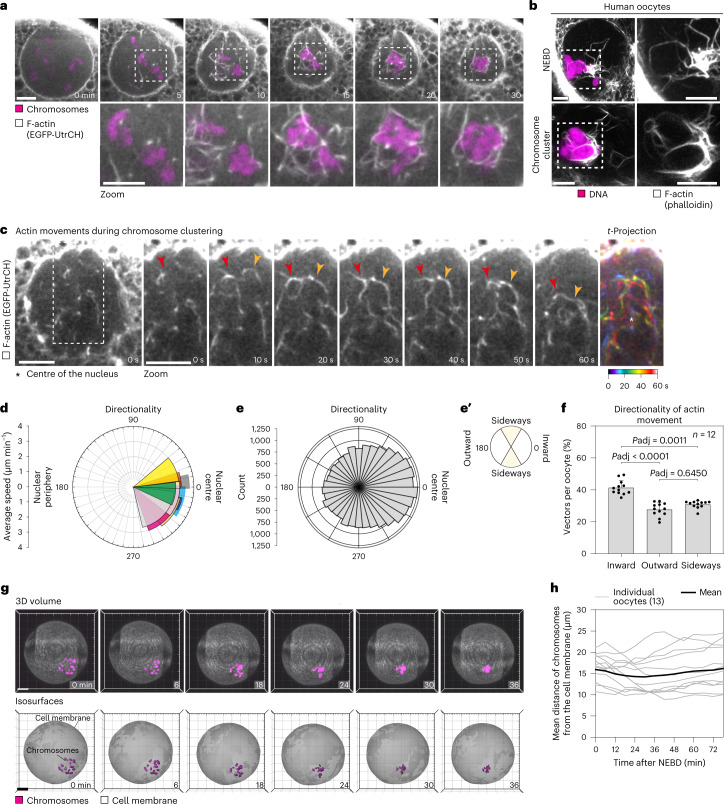
Chromosome clustering is driven by a dynamic network of interlinked actin cables. **a**, Porcine oocyte expressing the EGFP-UtrCH (F-actin) and H2B-mCherry (chromosomes). Outlined regions are magnified below. **b**, Immunofluorescence airyscan images of human oocytes shortly after NEBD (*Z*-projection, 11 sections every 0.19 µm) and after chromosome cluster formation. Representative examples of 27 immunolabelled human oocytes. White, F-actin (phalloidin); magenta, DNA (Hoechst); Lamin A/C, not shown; α-tubulin, not shown. Outlined regions are magnified on the right-hand side. Scale bar, 5 µm. **c**, Porcine oocytes expressing EGFP-Lamin B1 (not shown) and mScarlet-UtrCH (F-actin). Time series of the highlighted region is shown on the right-hand side. Coloured arrowheads point to actin cables in the nuclear region. White asterisk in *t*-projection panel shows direction to the nuclear centre. **d**, Polar plot showing directionality of velocity vectors detected in the nuclear region based on actin signal. Plot shows mean directions and the 95% confidence intervals of the velocity vectors and the average speed of those velocity vectors. **e**, Polar plot showing the distribution of velocity vectors detected in the nuclear region based on actin signal. **e**′, Directional velocity vectors as shown in **e** divided into four categories based on their orientation in respect to the nuclear centre (0°). Each category encompasses 120°. **f**, Bar plot showing the percentage of velocity vectors per oocyte in each category based on categorization in **e′**. Data are presented as mean ± s.d. Values from individual oocytes are shown as dots. Statistical test: Kruskal–Wallis one-way ANOVA test with Dunn’s multiple comparisons test. *P* values indicated as *P*adj above the box plot. **g**, Porcine oocytes expressing GFP with myristoylation signal (MyrGFP) (cell membrane) and H2B-mScarlet (chromosomes). Scale bar, 20 µm. Top: Three-dimensional (3D) volume of an entire porcine oocyte (Imaris, Bitplane). Bottom: isosurface rendering (Imaris, Bitplane) of the cell membrane and chromosomes. **h**, Line graph showing mean distance of chromosomes from the cell membrane. Scale bar, 10 µm unless otherwise specified. Time, minutes (min) or seconds (s) from NEBD. Number of oocytes in brackets. Data from three (**a**), four (**c**–**f**) or two (**g** and **h**) independent experiments. Source numerical data are available in source data. Source data

**Extended Data Fig. 4:**
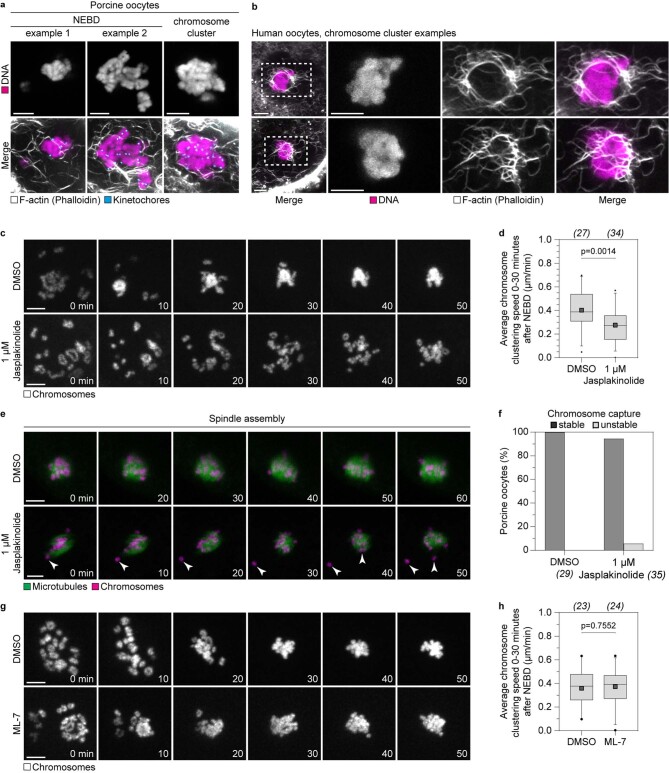
Chromosome clustering is driven by dynamic actin cables. **a**, Immunofluorescence images of porcine oocytes shortly after NEBD (z-projections, example 1: 14 sections, example 2: 11 sections every 0.19 µm) and after chromosome cluster formation (z-projection, 14 sections every 0.19 µm). White, F-actin (phalloidin); cyan, kinetochores (ACA); magenta, DNA (Hoechst). Scale bar, 5 µm. **b**, Immunofluorescence images of human oocytes after chromosome cluster formation. White, F-actin (phalloidin); magenta, DNA (Hoechst); alpha-tubulin (not shown); Lamin A/C (not shown). Outlined regions are magnified on the right. Z-projections, 6 (top) or 5 sections (bottom) every 0.19 µm. Scale bar, 5 µm. Representative examples from 27 oocytes. **c**, DMSO- or 1 µM jasplakinolide-treated porcine oocytes expressing H2B-mCherry (chromosomes) and EGFP-MAP4 (not shown). Z-projections, 12 sections every 3 µm. **d**, Quantification of average chromosome clustering speed between 0–30 min after NEBD in DMSO- and 1 µM jasplakinolide-treated porcine oocytes. Statistical test: two-tailed unpaired *t*-test (p = 0.0014). **e**, DMSO- or 1 µM jasplakinolide-treated porcine oocytes expressing H2B-mCherry (chromosomes) and EGFP-MAP4 (microtubules). Z-projections, 15 sections every 3 µm. White arrowheads point to uncaptured chromosomes. **f**, Quantification of the percentage of porcine oocytes with all chromosomes captured during spindle assembly. **g**, DMSO- or ML-7- treated porcine oocytes expressing H2B-mCherry (chromosomes). Z-projections of 10 (top) or 8 sections (bottom) every 3 µm. **h**, Quantification of average chromosome clustering speed between 0–30 min after NEBD in DMSO- and ML-7-treated porcine oocytes. Statistical test: two-tailed unpaired *t-*test (p = 0.7552). Scale bar, 10 µm unless otherwise specified. Time, minutes (min) after NEBD (**c**,**g**) or from the onset of the image sequence (**e**). Number of oocytes in brackets. Data from three independent experiments unless otherwise specified. Box plots show median (horizontal black line), mean (small black squares), 25th and 75th percentiles (boxes), 5th and 95th percentiles (whiskers), 1st and 99th percentiles (dots). Source numerical data are available in source data. Source data

To visualize actin filaments as they drive chromosome clustering, we expressed the F-actin probe EGFP-UtrCH (enhanced green fluorescent protein fused to the calponin homology domain of human Utrophin)^28,29^ in live porcine oocytes. Labelling of F-actin revealed the transient formation of interlinked actin cables in the nuclear region that persisted until around 60 min after NEBD, thus overlapping with the rapid phase of chromosome clustering (Fig. 4a and Supplementary Video 5). The actin cables were closely associated with chromosomes and contracted as the chromosomes clustered, appearing to move the chromosomes towards each other (Fig. 4a and Supplementary Video 5). Ultimately, the actin cables formed a structure resembling a shell that encapsulated the clustered chromosomes (Fig. 4a). Importantly, the actin cables were also detectable in fixed oocytes stained with phalloidin and are thus not an artefact caused by the EGFP-UtrCH probe (Extended Data Fig. 4a). Similar to porcine oocytes, fixed human oocytes displayed prominent actin cables encapsulating chromosomes shortly after NEBD (Fig. 4b and Extended Data Fig. 4b).

High-temporal-resolution microscopy revealed that new actin cables appeared primarily in the periphery of the nuclear region, from where they migrated inwards towards the centre of the nucleus (Fig. 4c–e and Supplementary Video 5). The assembly and migration of actin cables repeated multiple times after NEBD (Supplementary Video 5). The actin cables did not form simultaneously along the entire periphery of the nuclear region, but rather sequentially in different regions of the nuclear periphery (Supplementary Video 5). The actin filaments were significantly more likely to move inwards than outwards or sideways (Fig. 4f). The chromosomes, too, moved towards the centre of the former nucleus, with their mean distance from the cell membrane remaining largely constant during clustering (Fig. 4g,h).

To confirm that dynamic actin filaments are required for efficient chromosome clustering, we treated porcine oocytes with the actin-stabilizing drug jasplakinolide. Jasplakinolide treatment significantly decreased the speed of chromosome movement during 0–30 min after NEBD (Extended Data Fig. 4c,d) and interfered with chromosome capture in some oocytes (Extended Data Fig. 4e,f). Together, these data establish that dynamic actin filaments are required for efficient chromosome clustering.

Non-muscle myosin II (nm-myosin II) is a main driver of subcellular actin-dependent motility. In human mitotic cells, nm-myosin II is required for chromosome positioning after NEBD^16^. However, inhibition of nm-myosin II did not have an effect on chromosome clustering (Extended Data Fig. 4g,h), suggesting that nm-myosin II is dispensable for chromosome movements after NEBD in porcine oocytes.

### Actin cables interact with kinetochores during clustering

**Fig. 5:**
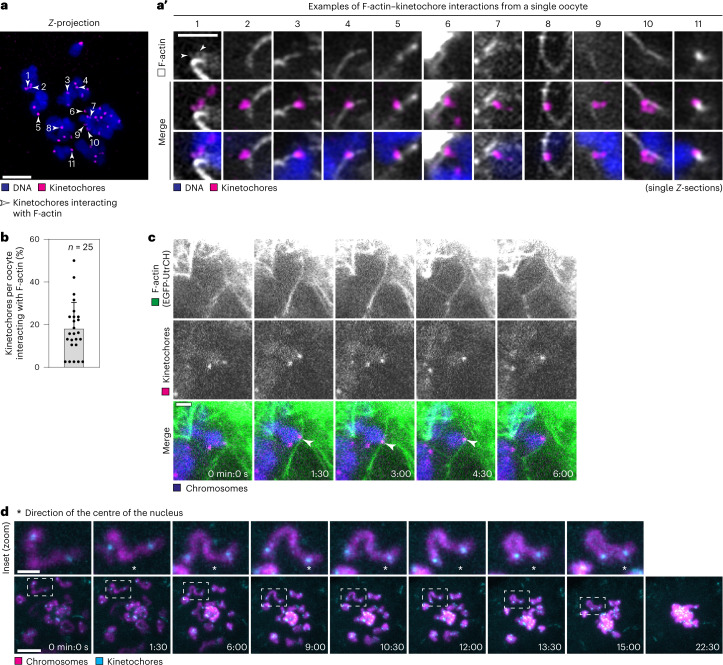
Actin cables interact with kinetochores during chromosome clustering. **a**, Immunofluorescence airyscan image of a porcine oocyte shortly after NEBD (*Z*-projection, 83 sections every 0.19 µm). White, F-actin (phalloidin, not shown); magenta, kinetochores (ACA); blue, DNA (Hoechst). Scale bar, 5 µm. White arrowheads point at kinetochores that were classified as actin-interacting. Each kinetochore is numbered. **a**′, Overview of the kinetochores highlighted in **a** with F-actin (phalloidin, white); magenta, kinetochores (ACA); blue, DNA (Hoechst). Scale bar, 2 µm. White arrowheads point at two actin spikes in the first image panel. **b**, Bar graph showing percentage of kinetochores per porcine oocyte interacting with F-actin as shown in **a** and **a′**. Data are presented as mean ± s.d. Values from individual oocytes are shown as dots. **c**, Time series of porcine oocyte expressing EGFP-UtrCH (F-actin), mScarlet-hCenpC (kinetochores) and H2B-Snap-SiR-647 (chromosomes). White arrowheads point to actin–kinetochore interactions. Scale bar, 2 µm. **d**, Time series of a porcine oocyte expressing mClover3-hCenpC (kinetochores) and H2B-mCherry (chromosomes). *Z*-projection, 22 sections every 1.5 µm. Scale bar, 10 µm. Highlighted region is magnified above. Scale bar, 2 µm. Asterisk shows the direction of the nuclear centre. Time, minutes:seconds (min:s) from NEBD. Number of oocytes indicated with *n*. Data from three or two independent experiments. Source numerical data are available in source data. Source data

**Extended Data Fig. 5:**
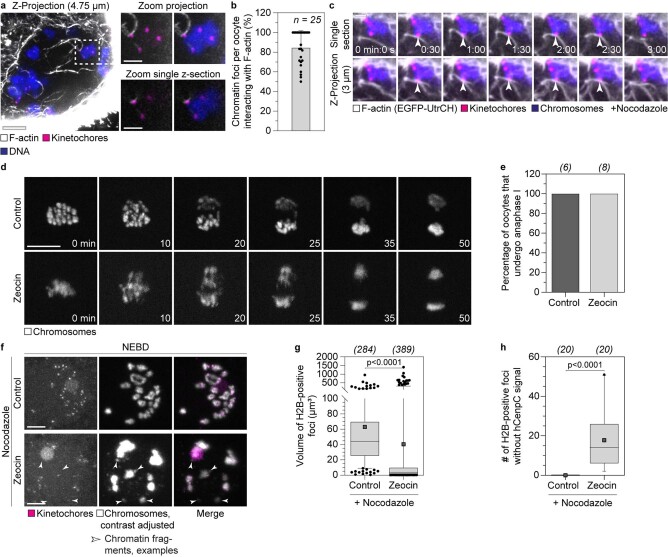
Actin cables closely interact with chromosome arms and kinetochores. **a**, Immunofluorescence airyscan images of porcine oocytes shortly after NEBD. Z-projection of 4.75 µm (sections every 0.19 µm). White, F-actin (phalloidin); magenta, kinetochores (ACA); blue, DNA (Hoechst). Scale bar, 5 µm. Highlighted region is magnified on the right. Scale bar, 2 µm. **b**, Bar plot showing percentage of chromatin foci per oocyte that closely interact with F-actin. Plot shows mean values +/− s.d., individual measurements shown as dots. **c**, Porcine oocyte expressing EGFP-UtrCH (F-actin), mScarlet-Cenp-C (kinetochores) and H2B-Snap-SiR-647 (chromosomes) in the presence of nocodazole. Bottom, z-projection of 3 µm (sections every 0.75 µm). White arrowheads point to actin-kinetochore interactions. Scale bar, 2 µm. **d**, Anaphase onset in control and zeocin-treated porcine oocytes expressing H2B-mCherry (chromosomes) and mClover3-hCenpC (not shown). Time, minutes after anaphase onset. Z-projection, 75 sections every 2 µm. Scale bar, 17.3 µm. Data from several biological replicates. **e**, Bar plot showing the percentage of control and zeocin-treated oocytes that progress through anaphase I. **f**, Chromatin configuration at NEBD in control and zeocin-treated porcine oocytes expressing H2B-mCherry (chromosomes) and mClover3-hCenpC (kinetochores). Z-projections, 15 sections every 3 µm. White arrowheads point to selected chromatin fragments. Scale bar, 10 µm. **g**, Box plot showing volume of H2B-positive foci in control and zeocin-treated oocytes. Statistical test: two-tailed Mann-Whitney test (p<0.0001). **h**, Box plot showing number of H2B-positive foci without hCenpC signal in control and zeocin-treated oocytes. Statistical test: two-tailed unpaired *t*-test with Welch’s correction (p<0.0001). Time, minutes (min) or minutes:seconds (min:s). Number of oocytes in brackets, indicated with *n*, or number of detected chromatin foci (**g**). Data from two independent experiments, unless otherwise specified. Box plots (**g-h**) show median (horizontal black line), mean (small black squares), 25th and 75th percentiles (boxes), 5th and 95th percentiles (whiskers), 1st and 99th percentiles (dots). Source numerical data are available in source data. Source data

Next, we investigated how the chromosomes interact with the actin cables during clustering in porcine oocytes. Strikingly, up to 50% of kinetochores per oocyte interacted with actin filaments, with an average of 18% of kinetochores per oocyte, as evident from fixing oocytes at random stages of chromosome clustering (Fig. 5a,b and Extended Data Fig. 5a). We also observed close contact between actin and chromosome arms (Fig. 4a,b and Extended Data Fig. 4a,b). On average, 85% of chromatin foci had at least one actin cable on their surface in fixed porcine oocytes (Extended Data Fig. 5b).

Three-colour imaging of chromatin, kinetochores and actin revealed that kinetochores and actin cables moved together in the same direction while they interacted (Fig. 5c and Supplementary Video 6). Kinetochore–actin interactions persisted for ~6–7 min in several cases, thus spanning a considerable fraction of the chromosome clustering period (Fig. 5c). Coordinated kinetochore–actin movements remained detectable in oocytes treated with the microtubule-depolymerizing drug nocodazole (Extended Data Fig. 5c and Supplementary Video 6), indicating that the interaction and movements are independent of microtubules. Additionally, we observed examples of chromosomes whose kinetochores re-oriented towards the nuclear centre during the actin-dependent phase of chromosome clustering, indicative of kinetochore-led chromosome movements (Fig. 5d and Supplementary Video 7).

**Fig. 6:**
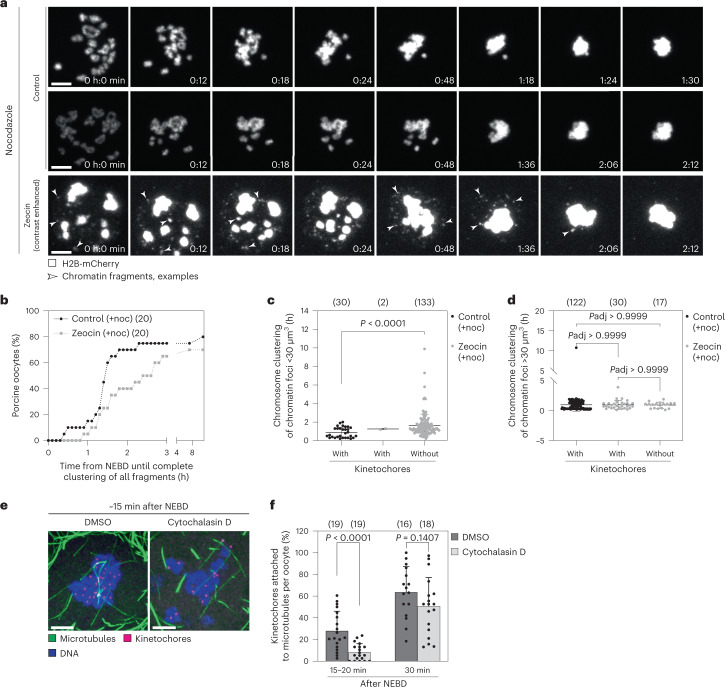
Smaller chromatin fragments without kinetochores cannot be clustered efficiently by actin. **a**, Time series of control and zeocin-treated porcine oocytes expressing H2B-mCherry (chromosomes) and mScarlet-hCenpC (kinetochores, not shown). Both groups were imaged in nocodazole containing medium to ensure that only microtubule-independent movements were analysed. *Z*-projections, 15 sections every 3 µm. White arrowheads point to examples of chromatin fragments. Scale bar, 10 µm. Data from two independent experiments. **b**, Line graph showing the percentage of control or zeocin-treated porcine oocytes that completely cluster all chromosomes and chromatin fragments as shown in **a**. Number of oocytes in brackets. **c**, Scatter dot plot showing time of clustering of individual chromatin fragments smaller than 30 µm^3^ in control and zeocin-treated porcine oocytes as shown in **a**. Chromatin foci with kinetochores from the control group and chromatin foci without kinetochores from the experimental group were compared using two-tailed Mann–Whitney test (*P* < 0.0001). **d**, Scatter dot plot showing time of clustering of individual chromatin fragments larger than 30 µm^3^ in control and zeocin-treated porcine oocytes as shown in **a**. Statistical test: Kruskal–Wallis one-way ANOVA test with Dunn’s multiple comparisons test. **e**, Immunofluorescence airyscan images of porcine oocytes fixed ~15 min after NEBD treated with DMSO or cytochalasin D (*Z*-projection, control: 40 sections every 0.19 µm, cytochalasin D-treated oocyte: 20 sections every 0.19 µm). Green, microtubules (α-tubulin); magenta, kinetochores (ACA); blue, DNA (Hoechst). Scale bar, 5 µm. **f**, Bar graph shows percentage of kinetochores attached to microtubules per oocyte in control and cytochalasin D-treated groups. Data from four independent experiments. Statistical test: two-tailed unpaired *t*-test. Data are presented as mean ± s.d., individual values shown as dots (**c**, **d** and **f**). Number of oocytes in brackets. *P* values are indicated above the box/dot plots. Source numerical data are available in source data. Source data

Next, we examined whether chromosome clustering requires kinetochore–actin interactions, or whether chromosome arm–actin interactions are sufficient. Disrupting kinetochores by Trim-Away^30,31^ was not possible because this approach is inefficient for nuclear proteins^30^. Instead, we treated porcine oocytes with the DNA cleaving antibiotic zeocin to generate kinetochore-containing and kinetochore-free chromosome fragments, following a previously established experimental strategy^12^. Oocytes treated with zeocin progressed into anaphase (Extended Data Fig. 5d,e), showing that zeocin does not block oocyte maturation. To evaluate microtubule-independent movements, we co-treated oocytes with nocodazole (Extended Data Fig. 5f–h). Small kinetochore-free chromosome fragments (<30 μm^3^) clustered more slowly than small kinetochore-containing chromosomes (<30 μm^3^) in control oocytes (Fig. 6a–c). In contrast, kinetochore-containing and kinetochore-free fragments that were larger than 30 µm^3^ clustered with similar efficiency (Fig. 6d). This shows that larger chromosomes are efficiently moved by chromosome arm–actin interactions. Smaller, kinetochore-free fragments, however, cannot be clustered efficiently.

**Extended Data Fig. 6:**
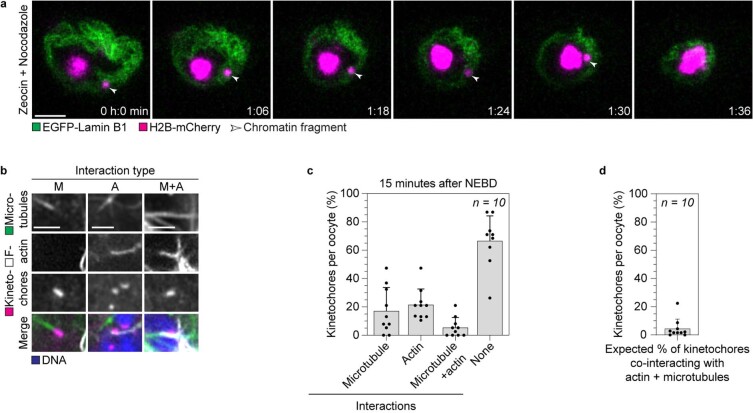
Actin-interacting kinetochores can bind microtubules. **a**, Porcine oocytes expressing EGFP-Lamin B1 and H2B-mCherry (chromosomes) treated with zeocin and nocodazole. Z-projection, 3 sections every 2 µm. White arrowheads point to a chromosome fragment that clusters as the nuclear lamina constricts. Scale bar, 10 µm. Time, hours:minutes (h:min). **b**, Immunofluorescence airyscan images of porcine oocytes shortly after NEBD. Blue, DNA (Hoechst); green, microtubules (alpha-tubulin); white, F-actin (phalloidin); magenta, kinetochores (ACA). Scale bar, 2 µm. **c**, Bar plot showing percentage of kinetochores interacting with microtubules (M), actin cables (A), or both simultaneously (M+A) as shown in **b**. **d**, Bar plot showing expected percentage of kinetochore interacting with actin and microtubules, based on the observed frequency of individual actin-kinetochore, and actin-microtubule interactions as shown in **c**. Data from two independent experiments. Data are presented as mean values +/− s.d., individual measurements shown as dots (**c**,**d**). Source numerical data are available in source data. Source data

The small kinetochore-free chromosome fragments eventually coalesced with a prominent delay (Fig. 6a) that correlated with the compaction of the nuclear lamina (Extended Data Fig. 6a), consistent with our hypothesis that the compacting nuclear lamina can coalesce chromosomes that failed to be clustered by other mechanisms, as described above. We infer that kinetochore–actin interactions are required to accelerate the clustering of small chromosomes.

### Actin-interacting kinetochores can bind microtubules

In starfish oocytes, the actin nucleation factor Arp2/3 assembles actin patches on chromosomes during chromosome migration, which sterically prevent the premature association of kinetochores with microtubules^12^. Similar actin patches do not exist in porcine oocytes (Figs. 4a,b and 5a and Extended Data Fig. 5a). Nevertheless, we considered that the actin cables that interact with kinetochores might also inhibit the association with microtubules. However, we found that the opposite was the case, the attachment of microtubules to kinetochores was lower in cells treated with cytochalasin D compared with DMSO-treated control oocytes (Fig. 6e,f). Additionally, co-staining of actin and microtubules in control oocytes revealed a fraction of kinetochores that was attached to microtubules while also interacting with actin (Extended Data Fig. 6b,c). The fraction size corresponded roughly to what was expected for random overlap between actin- and microtubule-interacting kinetochores (Extended Data Fig. 6d). Thus, kinetochore–actin interactions do not block the formation of kinetochore–microtubule attachments in porcine oocytes.

### Formin-2 and Spire mediate actin cable assembly

**Extended Data Fig. 7:**
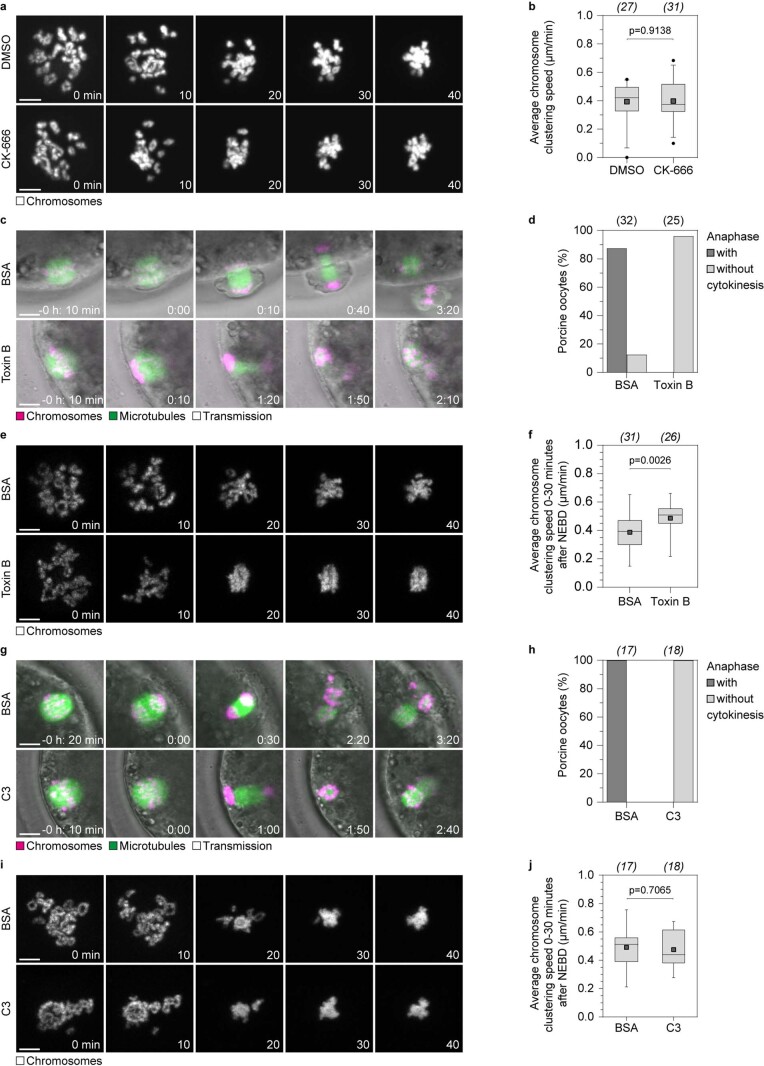
Functional analysis of Formin-mediated actin nucleation during chromosome clustering in porcine oocytes. **a**, Live porcine oocytes expressing H2B-mCherry (chromosomes) treated with DMSO or 200 µM CK-666. Z-projections, 15 sections every 3 µm. **b**, Quantification of average chromosome clustering speed between 0–30 min after NEBD in DMSO- and 200 µM CK-666-treated porcine oocytes. Statistical test: two-tailed Mann-Whitney test (p = 0.9138). **c**, Live porcine oocytes expressing H2B-mCherry (chromosomes) and EGFP-MAP4 (microtubules) upon microinjection with toxin B (bottom) or bovine serum albumin (BSA) (top). Z-projections, 9 sections every 3 µm. **d**, Quantification of completion or failure of cytokinesis in porcine oocytes microinjected with toxin B or BSA. **e**, Live porcine oocytes expressing H2B-mCherry (chromosomes) upon microinjection with toxin B (bottom) or BSA (top). Z-projections, 11 sections every 3 µm. **f**, Quantification of average chromosome clustering speed between 0–30 min after NEBD in BSA or toxin B microinjected porcine oocytes. Statistical test: two-tailed unpaired *t*-test (p = 0.0026). **g**, Live porcine oocytes expressing H2B-mCherry (chromosomes) and EGFP-MAP4 (microtubules) upon microinjection with C3 (bottom) or BSA (top). Z-projections, 9 sections every 3 µm. **h**, Quantification of completion or failure of cytokinesis in porcine oocytes microinjected with C3 or BSA. **i**, Live porcine oocytes expressing H2B-mCherry (chromosomes) and EGFP-MAP4 (not shown) upon microinjection with C3 (bottom) or BSA (top). Z-projections, 11 sections every 3 µm. **j**, Quantification of average chromosome clustering speed between 0–30 min after NEBD in BSA or C3 microinjected porcine oocytes. Statistical test: two-tailed unpaired *t*-test (p = 0.7065). Scale bar, 10 µm. Time, hours:minutes (h:min) from anaphase onset (**c,g**) or minutes (min) from NEBD (**a,e,i**). Number of oocytes in brackets. Data from three (**a–f**) or two independent experiments (**g–j**). Box plots (**b,f,j**) show median (horizontal black line), mean (small black squares), 25th and 75th percentiles (boxes), 5th and 95th percentiles (whiskers), 1st and 99th percentiles (dots). Source numerical data are available in source data. Source data

Next, we aimed to identify the F-actin assembly factors that drive the formation of actin cables in porcine oocytes. The cables were long and unbranched in nature (Figs. 4a–c and 5a,c), suggesting that their assembly is probably driven by formins rather than the Arp2/3 complex, which typically generates dense actin meshworks^12,32,33^. Indeed, treating porcine oocytes with the Arp2/3 inhibitor CK-666 had no effect on the speed of chromosome clustering between 0 min and 30 min post-NEBD (Extended Data Fig. 7a,b), indicating that Arp2/3 is dispensable for chromosome clustering.

Diaphanous-related formins assemble unbranched actin structures and rely on activation by Rho-GTPases^34,35^. To test their potential involvement in chromosome clustering, we inhibited Rho-GTPases with toxin B. We observed a full block of Rho-GTPase-dependent cytokinesis during polar body extrusion (Extended Data Fig. 7c,d), indicating efficient inhibition of Rho-GTPase activity. However, the speed of chromosome clustering was not decreased (Extended Data Fig. 7e,f). Similarly, inhibition of the Rho-GTPases RhoA, RhoB and RhoC with exoenzyme C3 fully blocked cytokinesis during polar body extrusion (Extended Data Fig. 7g,h), but did not decrease the speed of chromosome clustering (Extended Data Fig. 7i,j). We infer that chromosome clustering is not driven by diaphanous-related formins.

**Extended Data Fig. 8:**
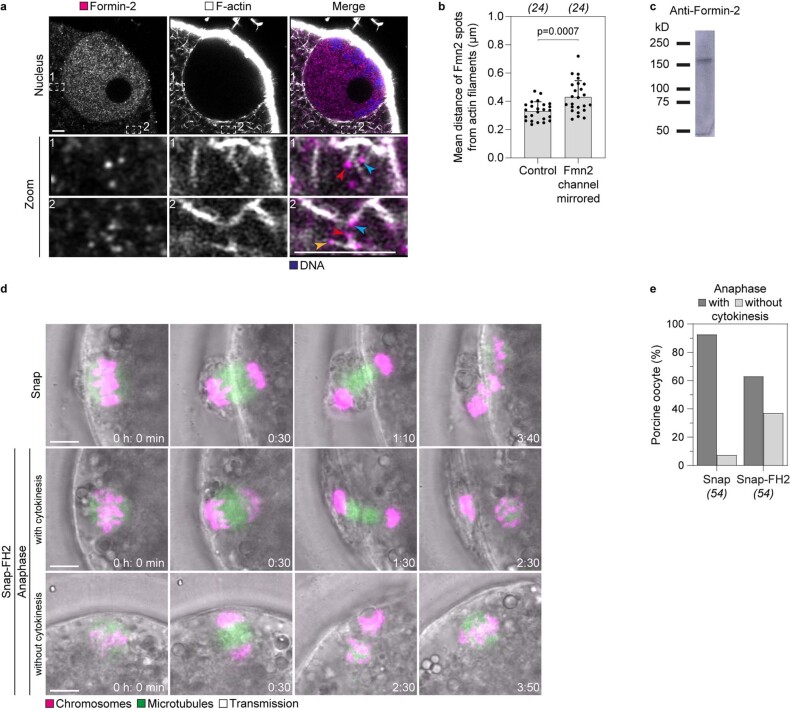
The actin nucleation factors Formin-2 is enriched in the nucleus of porcine oocytes. **a**, Immunofluorescence airyscan image of a porcine oocyte before NEBD. White, F-actin (phalloidin); magenta, Formin-2. Scale bar, 5 µm. Outlined regions are magnified below. Colored arrowheads point to Formin-2 foci on actin filaments. **b**, Bar plot showing mean distance of Formin-2 (Fmn2) spots from actin filaments in cytoplasm of porcine oocytes. Data are presented as mean values +/− s.d., individual values shown as dots. Statistical test: two-tailed unpaired *t*-test with Welch’s correction (p = 0.0007). **c**, Western blot from 30 porcine oocytes stained with anti-Formin-2 antibody. **d**, Live porcine oocytes expressing H2B-mCherry (chromosomes), EGFP-MAP4 (microtubules) and Snap or Snap-FH2 progressing through anaphase with or without successful cytokinesis. Z-projections, 9 sections every 3 µm. **e**, Quantification of completion or failure of cytokinesis in porcine oocytes expressing Snap or Snap-FH2 as shown in **d**. Scale bar, 10 µm. Time, minutes (min) from NEBD or hours:minutes (h:min) from anaphase onset. Number of oocytes in brackets. Data from three independent experiments. Source numerical data and unprocessed blots are available in source data. Source data

Formin-2 is well established to assemble actin structures in mouse oocytes and zygotes^29,36–39^. We found that Formin-2 was enriched in the nucleus of immunostained porcine oocytes (Extended Data Fig. 8a), making it an attractive candidate for the assembly of the actin cables. Formin-2 co-localized with actin filaments in the cytoplasm, suggesting that the antibody used for the immunofluorescence analysis was specific (Extended Data Fig. 8b). Endogenous expression of Formin-2 in porcine oocytes was further confirmed by western blot (Extended Data Fig. 8c).

**Fig. 7:**
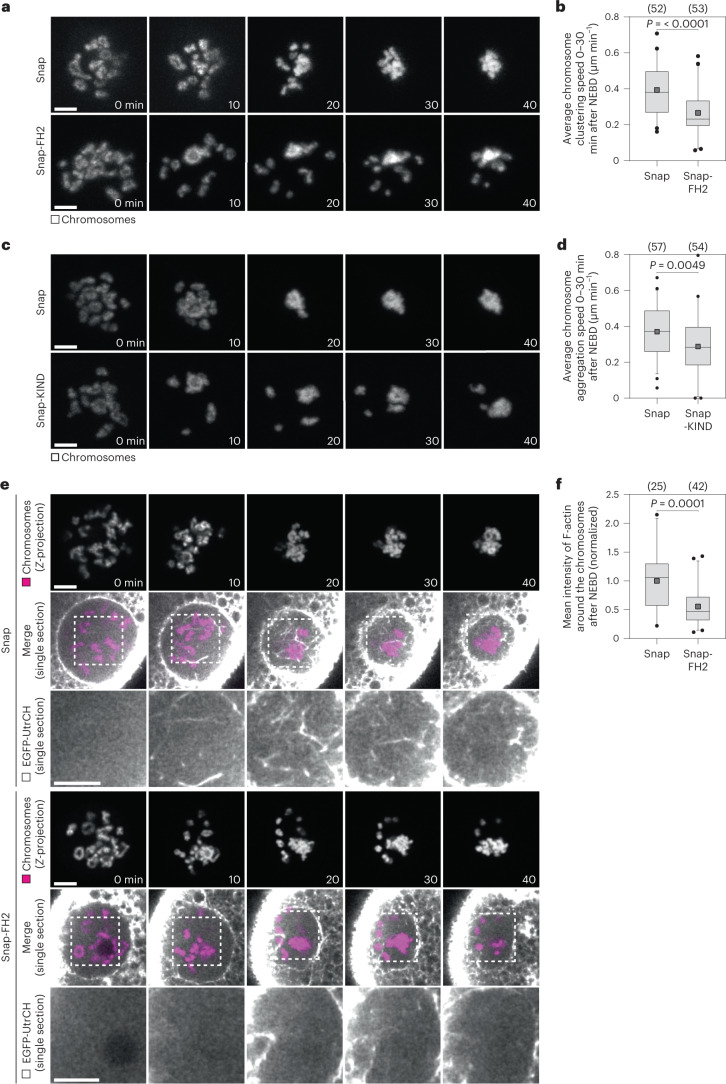
The actin nucleation factors Formin-2 and Spire mediate actin cable assembly. **a**, Porcine oocytes expressing H2B-mCherry (chromosomes), EGFP-MAP4 (not shown) and Snap or Snap-FH2. *Z*-projections, 11 sections every 3 µm. **b**, Quantification of average chromosome clustering speed between 0 min and 30 min after NEBD in porcine oocytes expressing Snap or Snap-FH2 as shown in **a**. Statistical test: two-tailed Mann–Whitney test (*P* = < 0.0001). **c**, Live porcine oocytes expressing H2B-mCherry (chromosomes), EGFP-MAP4 (not shown) and Snap or Snap-KIND. *Z*-projections, 13 sections every 3 µm. **d**, Quantification of average chromosome clustering speed between 0 min and 30 min after NEBD in porcine oocytes expressing Snap or Snap-KIND as shown in **c**. Statistical test: two-tailed unpaired *t*-test (*P* = 0.0049). **e**, Porcine oocytes expressing EGFP-UtrCH (F-actin), H2B-mCherry (chromosomes) and Snap or Snap-FH2. Outlined regions are magnified bellow. Individual chromosome panels: *Z*-projection, four sections every 6 µm. **f**, Quantification of mean fluorescence intensity of F-actin filaments around the chromosomes after NEBD in porcine oocytes expressing Snap or Snap-FH2 as shown in **e**. Statistical test: two-tailed Mann–Whitney test (*P* = 0.0001). Scale bar, 10 µm unless otherwise specified. Time, minutes (min) from NEBD. Number of oocytes in brackets. Data from three (**a**–**d**) or two (**e** and **f**) independent experiments. Box plots show median (horizontal black line), mean (small black squares), 25th and 75th percentiles (boxes), 5th and 95th percentiles (whiskers) and 1st and 99th percentiles (dots). Source numerical data are available in source data. Source data

Formin-2-dependent actin nucleation can be inhibited by expression of FH2- and KIND-domain constructs, which block the interaction between Formin-2 and Spire, an actin assembly factor that cooperates with Formin-2 to form actin filaments^36,40–42^. Expression of FH2 and KIND in porcine oocytes significantly decreased the speed of chromosome clustering (Fig. 7a–d). Moreover, FH2 expression reduced the abundance of actin cables in the nuclear region (Fig. 7e,f). Interestingly, FH2 expression led to a partial block of cytokinesis during polar body extrusion, suggesting that Formin-2 and Spire are involved in polar body extrusion in porcine oocytes (Extended Data Fig. 8d,e). We conclude that the chromosome-interacting actin cables are assembled by Formin-2 and Spire.

### Microtubule loops transport residual unclustered chromosomes

**Fig. 8:**
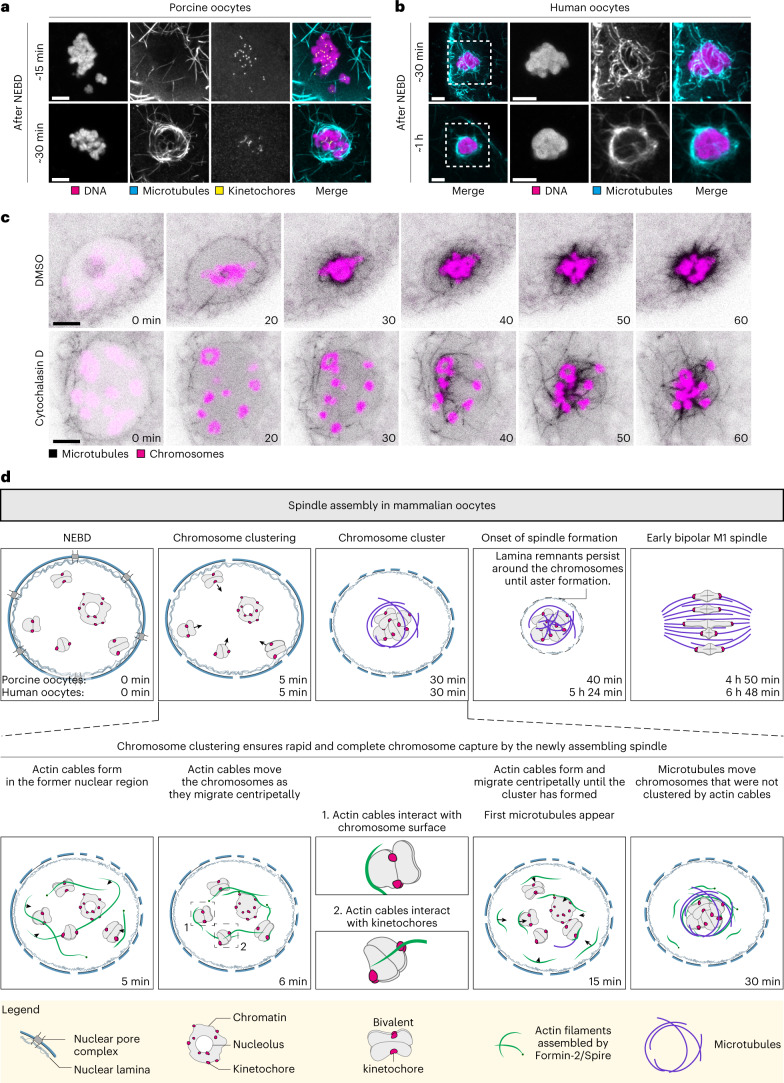
Stable microtubule loops transport chromosomes that fail to be clustered by F-actin. **a**, Immunofluorescence airyscan images of porcine oocytes fixed 15 or 30 minutes after NEBD. Cyan, microtubules (α-tubulin); yellow, kinetochores (ACA); magenta, DNA (Hoechst). Top: *Z*-projection, 25 sections every 0.19 µm. Bottom: *Z*-projection, seven sections every 0.19 µm. **b**, Immunofluorescence airyscan images of human oocytes fixed 30 min or 1 h after NEBD. Cyan, microtubules (α-tubulin); magenta, DNA (Hoechst); F-actin (not shown); Lamin A/C (not shown). Top: *Z*-projection, 12 sections every 0.19 µm. Representative examples of 21 immunolabelled human oocytes. **c**, Porcine oocytes expressing EGFP-MAP4 (microtubules) and H2B-mCherry (chromosomes) treated with DMSO or cytochalasin D. *Z*-projection, four sections every 3 µm. **d**, Schematic illustration of the spindle assembly (top) and chromosome clustering (bottom) in porcine and human oocytes. Legend is depicted at the bottom of the figure. Based on experimental data presented in this paper. Timing of human oocyte spindle assembly is described elsewhere^5^. Scale bars, 5 µm (**a** and **b**) and 10 µm (**c**). Time, minutes (min) from NEBD. Data from three independent experiments unless otherwise specified.

To investigate how microtubules transport the chromosomes that were not clustered by actin cables by 30 min post-NEBD, we performed high-resolution microscopy of fixed porcine oocytes. We observed a cage of microtubule loops that encapsulated the chromosome cluster 30 min post-NEBD (Fig. 8a and Supplementary Video 8). Similar to kinetochore-associated microtubules, these microtubule loops were not depolymerized by briefly placing the oocytes on ice (Extended Data Fig. 9 and Supplementary Video 9), indicating that the microtubule loops are stable. We detected a similar cage of microtubule loops in human oocytes as well, as early as 30 min after NEBD (Fig. 8b and Supplementary Video 8), suggesting that the formation of a microtubule cage is conserved between species.

The microtubule loops formed only during later stages of chromosome clustering (Fig. 8a,b). Thus, in the absence of F-actin, the microtubule loops transported the chromosomes with a notable delay (Figs. 3a and 8c). Live imaging of microtubules in cytochalasin D-treated porcine oocytes revealed that the chromosomes interacted with microtubule loops and clustered as the network contracted (Fig. 8c). These data establish that microtubule loops can move the chromosomes when F-actin-based clustering fails.

## Discussion

Here we report that chromosome clustering before spindle assembly in human and porcine oocytes prevents chromosome losses and facilitates the rapid and complete capture of all chromosomes by the assembling spindle. Our data indicate that chromosome clustering is safeguarded by multiple, partially redundant mechanisms, underscoring its importance (Fig. 8d).

Actin and microtubules were reported to drive chromosome transport between NEBD and the onset of spindle assembly in other species and cell types, where chromosome clusters do not form. Importantly, all of these mechanisms are distinct. The actomyosin network that forms around the disassembling nuclear envelope in human mitotic cells^16^ is located on the cytoplasmic side of the nuclear envelope^16^ and requires nm-myosin II activity to move the chromosomes^16^. Despite these mechanistic differences, the network in mitotic cells was proposed to reduce the nuclear volume in order to facilitate chromosome capture, suggesting functional similarities.

Starfish oocytes, unlike human and porcine oocytes, rapidly assemble a spindle with centrosomes and do not form a chromosome cluster. However, they too assemble an actin network in the nuclear region upon NEBD^13^. The actin meshwork does not interact specifically with kinetochores and has different dimensions from the actin cables that cluster chromosomes in mammalian oocytes as described here^12–14,43^. Moreover, in starfish oocytes, the chromosomes are coated by patches of Arp2/3-nucleated actin that shields chromosomes from microtubules^12^. In contrast, the chromosomes are neither coated nor shielded by actin in mammalian oocytes. Nevertheless, actin is essential for accurate progression through meiosis in all three species.

In *Xenopus* oocytes, a ~200-µm-wide array of microtubules moves across the nucleus to gather the chromosomes^15,44,45^. The microtubule loops we observed in porcine and human oocytes are morphologically very different from the microtubule array described in *Xenopus* oocytes^15,27^. However, it remains to be investigated whether there are similarities in the molecular players that drive the assembly of these two structures.

Taking all evidence together, there appears to be a conserved challenge that oocytes of various species must cope with. Their chromosomes are typically located within large nuclei, and they often lack centrosomes for spindle assembly, instead employing a chromosome-driven, Ran-dependent spindle assembly pathway. Work in mouse and *Xenopus* extracts shows that chromosome-driven spindle assembly results in the formation of multiple spindles in the presence of spatially separated chromosomal foci^46–48^. Oocytes thus have a conserved need to gather the chromosomes into one place, to prevent chromosome losses and to assemble a single spindle.

## Methods

### Ethics approval

The use of immature unfertilized human oocytes in this study has been approved by the UK’s National Research Ethics Service under the REC Reference 11/EE/0346 (IRAS Project ID 84952). The porcine oocytes used in this study were obtained from a local abattoir as a waste product of the slaughtering process, and as such do not require ethics approval for usage in Germany where the study was conducted.

### Primary porcine oocytes

All porcine oocytes were collected from pre-pubertal animals of female sex (exact age unknown). Porcine ovaries were obtained from a local abattoir and transported to the laboratory in a thermo-flask. Cumulus-oocyte complexes (COCs) were aspirated from 2–6-mm-large antral follicles using a 17-gauge needle affixed to a 1 ml disposable syringe. The aspired follicular fluid was collected into a 50 ml Falcon tube containing 2 ml of M2 medium with 1 mM dbcAMP. COCs were allowed to sediment for 15 min, and then washed extensively through four 35 mm Petri dishes, each containing 2 ml of pre-warmed M2 medium with 1 mM dbcAMP. Only fully grown oocytes with a homogeneous cytoplasm and at least three to five complete layers of compact cumulus cells were selected for the experiments. COCs were denuded using a series of transfer pipettes with a tip of defined diameter (175 µm, 145 µm and 135 µm) attached to an EZ-Grip denudation pipettor. Oocytes were cultured in M16 (5% CO_2_) or M2 medium at 38.5 °C. In most experiments, prophase arrest was maintained by supplementing the culture medium with dbcAMP. For experiments involving timed fixations and a few live cell imaging experiments specified below, cells were cultured for approximately 12 h in medium supplemented with 50 µM Butyrolactone I (Abcam, ab141520; 50 mM stock in ethanol). To induce the resumption of meiosis, oocytes were washed into dbcAMP/Butyrolactone I-free M2 medium.

### Primary human oocytes

All human oocytes obtained for this study were sourced from patients of female sex undergoing fertility treatments at Bourn Hall Clinic (Bourn, Cambridgeshire, UK) between 12 January 2017 and 24 July 2019 after having obtained fully informed consent. All donations were anonymous. Patients were not monetarily compensated for their donation. Ages of human oocyte donors from Fig. 1 and Extended Data Fig. 1a are listed in Supplementary Table 1. Ages of human oocyte donors for data shown in Figs. 4b and 8b and Extended Data Figs. 3b and 4b are listed in Supplementary Table 2. Fifty-six germinal vesicle (GV) stage oocytes from 39 donors were obtained for immunofluorescence analysis. All donors underwent ovarian stimulation for intracytoplasmic sperm injection. Only oocytes that were immature and hence unsuitable for intracytoplasmic sperm injection were donated to the study. None of the oocytes used in this study were freeze-thawed. Oocytes were cultured in G-MOPS medium (Vitrolife, 10129) supplemented with 10% FBS (GIBCO, 16000044) under mineral oil (Merck, 8012-95-1) at 37 °C. Only oocytes that appeared morphologically healthy and underwent NEBD within 24 h upon retrieval were included in the study. NEBD of the GV was observed using a Primo Vision Evo+ timelapse camera (Vitrolife) installed inside the incubator. Thirty-nine oocytes were fixed at defined timepoints around NEBD. NEBD was scored by the disappearance of the GV observed using the Primo Vision Evo+ and confirmed under the stereomicroscope. Thirteen cells were fixed in the GV stage, 25 cells were fixed within 30 min of NEBD and 1 cell was fixed 1 h after NEBD. The remaining 17 oocytes had NEBD shortly before they were obtained from the IVF clinic. All 56 human oocytes were co-stained with an anti-α-tubulin antibody (MCA78G, Bio-Rad; 1:3,000) to visualize microtubules, an anti-LaminA/C antibody (Sigma-Aldrich, SAB4200236; 1:50) to visualize the nuclear lamina, 100 µM Hoechst 33342 (Molecular Probes; 20 mM stock) to stain DNA, and phalloidin conjugated with Alexa Fluor 488 or Rhodamine (Invitrogen; 1:20) to label F-actin. Twenty-seven oocytes had visible actin cables encapsulating the chromosomes, 21 oocytes had a microtubule meshwork encapsulating the chromosomes, four had already started to assemble a spindle, two had a bipolar spindle and two cells were not well fixed as evident from a poor preservation of the actin and microtubule cytoskeleton following immunofluorescence analysis.

The images and graphs in Fig. 1 and Extended Data Fig. 1a were generated by analysing videos from 35 live human oocytes that were recorded as part of a previous study^5^. All cells included in the analysis underwent NEBD within 24 hours of retrieval, formed MI spindles and progressed through anaphase I.

### Expression constructs and mRNA synthesis

To generate the constructs for in vitro messenger RNA synthesis, previously published coding sequences were inserted into pGEMHE^49^ together with the miRFP^50^, mScarlet^51^, mClover3 (ref. ^52^) or SNAPf from pSNAPf (New England Biolabs) to generate pGEMHE-SNAPf, pGEMHE-RanWT-miRFP (Addgene #59750) (ref. ^53^), pGEMHE-RanT24N-miRFP (Addgene 61. ^53^), pGEMHE-H2B-SNAPf^54^, pGEMHE-SNAPf-FH2 (ref. ^36^), pGEMHE-SNAPf-KIND^36^, pGEMHE-mScarlet-UtrCH^28^ and pGEMHE-mClover3-hCenpC^55^. The expression constructs pGEMHE-H2B-mCherry^30^, pGEMHE-H2B-mScarlet^56^, pGEMHE-mEGFP-MAP4 (ref. ^30^), pGEMHE-EGFP-UtrCH^29^, pGEMHE-mScarlet-hCenpC^55^, pGEMHE-MyrGFP^29^ and pGEMHE-EGFP-Lamin B1 (ref. ^29^) were generated in previous studies. All mRNAs were synthesized with T7 polymerase (HiScribe T7 ARCA mRNA Kit) following the manufacturer’s instructions.

### Microinjection of porcine oocytes and transient protein expression

Porcine oocytes were microinjected with 2 pl of mRNAs as previously described^9^, with the exception that an ‘injection shelf’ was generated by assembling two coverslips around a spacer consisting of two layers of double-sided sticky tape (Scotch; 3 M, 136R2) to accommodate ~120–130-µm-large porcine oocytes. Oocytes were microinjected immediately after isolation and denudation. mRNAs were injected at the following concentrations: mEGFP-MAP4 at 26 ng µl^−1^, H2B-mCherry and H2B-mScarlet at 1–5 ng µl^−1^, H2B-SNAPf at 10–30 ng µl^−1^, mClover3-hCenpC and mScarlet-hCenpC at 20 ng µl^−1^, EGFP-UtrCH and mScarlet-UtrCH at 180–360 ng µl^−1^, RanWT-miRFP at 1.25 µg µl^−1^, RanT24N-miRFP at 2.5 µg µl^−1^, SNAPf-FH2 at 2 µg µl^−1^, SNAPf-KIND at 2.5 µg µl^−1^, SNAPf at 1–1.25 µg µl^−1^ and EGFP-Lamin B1 at 34.5 ng µl^−1^. In most experiments, oocytes were allowed to express mRNAs for 3–4 h before release into dbcAMP-free medium for imaging. For three-colour live imaging of F-actin, kinetochores and chromatin, high-temporal-resolution imaging of F-actin after NEBD and in experiments with zeocin; cells were incubated in 50 µM Butyrolactone I for 12–15 h before they were released into Butyrolactone I-free medium for imaging.

Exoenzyme C3 (Cytoskeleton, CT03) was reconstituted in embryo tested water to obtain a 10 µM stock. Oocytes were microinjected with 10 pl of 2.4 µM exoenzyme C3, or the equivalent amount of bovine serum albumin (BSA, Sigma, A3311). *Clostridium difficile* toxin B (Sigma-Aldrich, SML1153) was dissolved in RNase free water to obtain a 1.5 µM stock. Oocytes were microinjected with 10 pl of 100 nM toxin B, or an equivalent amount of BSA.

### Dextran labelling

Following the manufacturer’s instructions (MAN0001774|MP00143), 40 kDa amino dextran (Molecular Probes, D1861) was conjugated with Alexa Fluor 647 NHS Ester (Molecular Probes, A20006) in an equimolar reaction.

### Drug addition and washout

All drugs were prepared in hybridoma-grade DMSO (Sigma-Aldrich, D2650). To depolymerize microtubules, oocytes were treated with 10 µM nocodazole (Sigma-Aldrich, M1404). Nocodazole powder was freshly dissolved before each experiment to obtain a 15 mM stock. To depolymerize actin, oocytes were treated with 5 µg ml^−1^ cytochalasin D (Sigma-Aldrich, C8273; 25 mg ml^−1^ stock stored at −20 °C). To stabilize actin filaments, oocytes were treated with 1 µM jasplakinolide (Invitrogen, J7473; 1 mM stock stored at −20 °C). Nocodazole and cytochalasin D were added to dbcAMP-free M2 medium 30 min before imaging. Jasplakinolide was added to dbcAMP-free M2 medium 1 h 30 min before imaging. To wash out cytochalasin D and nocodazole, cells were washed through four 35 mm Petri dishes, each containing 2 ml of M2 medium without drugs. All drug washouts were done approximately 1–3 h after NEBD. The activity of Arp2/3 was inhibited using 200 µM CK-666 (Sigma-Aldrich, SML0006; 130 mM stock stored at −20 °C). Myosin light chain kinase was inhibited using 6 µM ML-7 (Sigma-Aldrich, 475880; 22 mM stock stored at −20 °C). CK-666 and ML-7 were added to dbcAMP-free M2 medium 1 h before imaging.

### Zeocin preparation

Zeocin powder (InvivoGen, ant-zn-1p) was dissolved in HEPES buffer at a stock concentration of 100 mg ml^−1^ (stock stored at 4 °C). To induce chromosome fragmentation, porcine oocytes were treated for 15 h with 500 µg ml^−1^ of zeocin diluted in M16 medium. To stop chromosome fragmentation, cells were transferred into zeocin-free M2 medium.

### Cold-mediated microtubule depolymerization assays

Porcine oocytes were incubated on ice for 7 min and immediately fixed and processed for immunofluorescence microscopy as described below.

### Immunofluorescence

To obtain oocytes at defined timepoints after NEBD, cells were monitored on a confocal microscope with transmission, or with a Primo Vision system. NEBD was determined as the moment when the nucleoli began to shrink.

Porcine and human oocytes were fixed in a solution containing 100 mM HEPES (pH 7.0, titrated with KOH; 1 M stock), 50 mM EGTA (pH 7.0, titrated with KOH; 0.5 M stock), 10 mM MgSO_4_ (1 M stock), 2% methanol-free formaldehyde (10% stock) and 0.5% Triton X-100 (10% stock; Sigma Aldrich, 93443) at 37 °C for 30 (porcine oocytes) or 60 min (human oocytes). Fixed oocytes were subsequently extracted in phosphate-buffered saline (PBS) supplemented with 0.5% Triton X-100 (PBT) overnight at 4 °C and thereafter blocked in PBT with 5% BSA (Fisher BioReagents BP1605-100, 11483823) (PBT-BSA) overnight at 4 °C. Porcine oocytes were then optically cleared as previously described^56^. All antibody incubations, F-actin and chromosome staining were performed in PBT-BSA. Primary antibodies were incubated overnight at 4 °C. Secondary antibody, fluorescently tagged phalloidin and Hoechst staining were performed for 1 h at room temperature. Primary antibodies used were rat anti-α-tubulin (MCA78G, Bio-Rad; 1:3,000), rabbit anti-Fmn2 (Atlas antibodies, HPA050649; 1:50), mouse anti-Lamin A/C (Sigma-Aldrich, SAB4200236; 1:50) and human anti-centromere antibody (ACA) (15–234, Antibodies Incorporated; 1:250). Secondary antibodies used were Alexa Fluor 488-, 564-, 568- or 647-conjugated anti-rat IgG, anti-rabbit IgG, anti-mouse IgG or anti-human IgG, all raised in goat or donkey (Molecular Probes; 1:400). DNA was stained with 100 µM Hoechst 33342 (Molecular Probes; 20 mM stock). F-actin was stained with phalloidin-Alexa Fluor 488 (Invitrogen; 1:20).

### Confocal microscopy

Confocal imaging was performed in ~20 µl of M2 medium (for live oocytes) or PBS with 1% polyvinylpyrrolidone (for fixed oocytes) under paraffin oil in a 35 mm dish with a #1.0 glass coverslip. Images were acquired with LSM800, LSM880 and LSM900 confocal laser scanning microscopes (Zeiss) equipped with an environmental incubator box and a 40× C-Apochromat 1.2 NA water-immersion objective. Live samples were imaged at 38.5 °C, and fixed at 25 °C. Automatic 3D tracking was implemented for time-lapse imaging with a temporal resolution between 1.5 min and 10 min using AutofocusScreen^57^ or MyPiC^58^ on the LSM880, or by using a customized sample tracking solution provided by ZEISS Microscopy on LSM800 and LSM900 microscopes. In all experiments, control and experimental groups were imaged together, under identical imaging conditions on the same microscope. For some images presented in the figures, noise was reduced with a Gaussian filter in ZEN (Zeiss). Airyscan images were acquired using the airyscan module on LSM800, LSM880 and LSM900 confocal laser scanning microscopes (Zeiss) and processed in ZEN (Zeiss) after acquisition. Care was taken that imaging conditions (laser power, pixel-dwell time and imaging frequency and detector gain) did not cause phototoxicity (for live imaging), photobleaching or saturation. The actin cortex was saturated in experiments where F-actin in the cytoplasm was imaged.

### Automatic detection of NEBD in porcine oocytes on a confocal microscope

The resumption of meiosis in porcine oocytes is asynchronous. Thus, to perform high-resolution spatial and temporal imaging of F-actin after NEBD, we designed a macro that allowed us to automatically detect NEBD.

To determine the timing of NEBD, we took advantage of a fluorescently labelled (Alexa Fluor 647 NHS Ester (succinimidyl ester); Invitrogen, A20006) 40 kDa dextran (Molecular Probes, A20006), which localizes to the cytoplasm in GV oocytes but upon NEBD quickly distributes within the nucleus. We recorded coarse resolution images of the dextran in the early stages of meiosis. The acquired images were read and analysed using the Bioformats^59^ package within a MATLAB Compiler Runtime Environment. To evaluate if NEBD had initiated, a sphere of 10 µm in diameter was placed at the centre of mass of the chromosome signal, and the average intensity of the dextran within this sphere was computed at every timepoint. A switch to high-resolution image acquisition was triggered when the intensity within the sphere became higher than a variable threshold. The threshold was determined for each oocyte at every timepoint individually, and was based on the median and the standard deviation of previous measurements. The script keeps the time interval for each imaged oocyte as the image acquisition interval set in the Zen software (Zeiss). The script was designed to work with the Zen Blue (Zeiss) image acquisition software.

### Light sheet microscopy

Light sheet imaging was performed on an LS1 Live light sheet microscope system by Viventis. The illumination optics consist of two opposing Nikon CFI Plan Fluor air illumination objectives. We used a Nikon CFI75 Apochromat 25xC W NA 1.1 water immersion objective with switchable magnification between 18.75× and 37.5× (field of view 700 µm and 340 µm, respectively) as detection objective. The microscope had a fast motorized six-position filter wheel with emission filters in front of the camera. The following laser lines and emission filters were used:

- Laser 488 nm, 60 mW diode laser, Semrock FF03-525/50-25 emission filter
- Laser 515 nm, 50 mW diode laser, Semrock FF01-539/30-25 emission filter
- Laser 561 nm, 50 mW DPSS laser, Semrock FF01-523/610-25 emission filter
- Laser 638 nm, 100 mW diode laser, Chroma ZET405/488/561/640 m emission filter.

The camera used was an Andor Zyla 4.2 PLUS USB 3 sCMOS.

### Immunoblotting

Thirty porcine GV oocytes per lane were washed in PBS and resuspended in 5 µl of PBS. NuPAGE LDS sample buffer (4×) (Thermo Fisher Scientific, NP0008) with 100 mM dithiothreitol was then added to the sample to a final 1× concentration, and the mixture was immediately snap-frozen in liquid nitrogen. Samples were thawed and frozen thrice more, followed by a 5 min incubation at 95 °C. Samples were resolved on a 10-well NuPAGE Tris-acetate protein gel of 1.0 mm thickness (Thermo Fisher Scientific, EA0375BOX) with NuPAGE Tris-Acetate SDS Running Buffer (Thermo Fisher Scientific, LA0041). Proteins were transferred onto an 0.45 µm Invitrolon PVDF Filter Paper Sandwich (Invitrogen, LC2005) with a NuPAGE Transfer Buffer (Thermo Fisher Scientific, NP00061) at 100 V for 1.5 h on ice. Blocking and antibody incubations were performed in PBS with 5% skimmed milk (w/v) (Sigma-Aldrich, LC2005) and 0.1% Tween-20. Primary antibodies were incubated overnight at 4 °C. We used rabbit anti-Fmn2 (Atlas antibodies, HPA05064; 1:1,000) as primary antibody and goat anti-Rabbit IgG (H + L) Cross-Adsorbed, HRP (ThermoFisher, 31462; 1:10,000) as secondary. Blots were developed with Super Signal West Femto Maximum Sensitivity Substrate (Thermo Fisher Scientific, 34094) and imaged with an Amersham Imager 600 (GE Healthcare).

### General quantifications

Time-lapse videos of live porcine oocytes were analysed using Imaris 8.4.1 or Imaris 9.3.1 (Bitplane). NEBD in human and porcine oocytes was defined as the last time point before the nucleolus begins to visibly shrink/disappear.

### Automatic creation of chromosome isosurfaces in Imaris

To consistently reconstitute the chromosome isosurfaces in Imaris, we used a MATLAB script published elsewhere^56^.

### Automatic quantification of chromosome clustering speed

To calculate the speed of chromosome clustering, we first calculated the largest pairwise distance measured between all chromosomes for every timepoint. The change of the largest pairwise distance between the timepoints was then used to derive the speed of chromosome clustering.

To consistently and reliably quantify the largest pairwise distance between all chromosomes, we developed an in-house MATLAB script.

The script requires that isosurfaces are defined as vertices connected into polygons, and hence works only with versions older than Imaris 9. The script computes distances between all pairs of vertices via the ‘pdist’ MATLAB function. The largest distance is then selected and depicted in Imaris as a pair of spots. The largest distance is also added as a custom statistic to the isosurface object. For usage within Imaris, the script was compiled for MATLAB Compiler Runtime v95.

### Automatic creation of a convex hull from 3D reconstituted chromosomes

To create a 3D convex hull, we used an in-house-developed MATLAB script. The script requires that isosurfaces are defined as vertices connected into polygons, and hence works only with versions older than Imaris 9. The script uses the MATLAB function ‘convhulln’ to identify the subset of vertices of a given isosurface making up the facets of the corresponding convex hull of this isosurface. For usage within Imaris, the script was compiled for MATLAB Compiler Runtime v93.

### PIV analysis from high-temporal-resolution F-actin imaging after NEBD

To measure velocity vectors within the nuclear area, we first determined the boundaries of the nucleus based on EGFP-Lamin B1 signal. For this, the signal of the nuclear lamina was segmented using edge-aware local contrast enhancement followed by edge detection. The resulting fragmented and noisy segmentation was translated to polar coordinates and the outliers were removed. The centre of the polar coordinate system was defined by the centre of mass of the segmented nuclear lamina. Closing of the membrane was achieved by fitting a smoothing spline to the coordinates theta and rho of the remaining pixels. The smoothing spline was expanded by 1.5 μm or 2.5 μm per direction to produce a segmented image of the nuclear lamina.

The analysis was performed in MATLAB using ‘localcontrast’, ‘edge’ (with Canny algorithm), ‘rmoutliers’ and ‘fit’ functions. The exact parameters for these functions had to be adjusted in a few cases of lower signal-to-noise ratio. To import the images into MATLAB R2018b, we used the toolbox Bioformats^59^.

To distinguish movement of the entire cell or nucleus from movement of actin, the images were stabilized with the option ‘translation’ using the Zeiss Zen function ‘time alignment’ on the signal of the nuclear lamina.

Actin velocities were detected using PIVlab^60^ in MATLAB R2018b on the stabilized images. The Wiener2 denoise and low pass filters with 5 pixel window size were used for pre-processing. The particle image velocimetry (PIV) algorithm used was ‘FFT window deformation’. The interrogation windows were reduced in three steps of 64, 32 and 24 pixels width. The correlation robustness was set to ‘extreme’. All options for post-processing were disabled, which resulted in weak filtering of the velocity vectors.

The direction of the velocity vectors was set relative to the centre of the nucleus. The centre of the nucleus was determined on the basis of the centre of mass of the widened smoothing spline. The segmentation of the nucleus was used to identify which vectors were located inside the nucleus, but not to mask the nuclear membrane for the PIV analysis.

Circular statistics were computed using the Circular Statistics Toolbox^61^. Mean directions and the 95% confidence intervals are based on the velocity vectors with speeds exceeding two pixels per frame or 1.8 µm min^−1^.

### Semi-automatic analysis of chromosome clustering upon chromosome fragmentation

H2B-positive foci were detected, counted and analysed using the surface function in Imaris 9.6.0 (Bitplane). Foci were classified manually as kinetochore-containing or kinetochore-free on the basis of the presence or absence of the hCenpC signal, respectively. The total time required for complete chromosome clustering was calculated as the time between NEBD and the first timepoint when only one H2B-positive surface was detected. Cells were scored as having completed chromosome clustering only if all the H2B-foci formed a single surface during the duration of imaging. Individual H2B foci with and without a kinetochore were tracked manually to estimate the time required for their clustering. Only foci that could be reliably tracked were analysed. Chromatin surrounding the nucleolus was excluded from the analysis of individual H2B foci.

### Co-localization analysis for Formin-2 and F-actin

The specificity of the anti Formin-2 antibody was tested using the statistic ‘Shortest distance to Surface’ computed using Imaris 9.7.2 and Imaris 9.3.0 by Bitplane.

For this purpose, spots were created once on the Formin-2 signal and once on the Formin-2 signal mirrored along the *x* axis. These spots were compared relative to a surface segmentation of the actin signal (Phalloidin). Before the analysis, a region of interest of 10 μm × 10 μm × 10 μm was manually cropped. The region of interest was placed so that it included neither the nucleus nor the very edge of the image. Around 100 spots were created per cell to improve comparability.

The surface on actin was created with a batch script previously published elsewhere^56^ using Otsu’s method for thresholding and ensuring the volume of the actin surface to be within 30 μm³ to 1,000 μm³.

### Manual quantification of the mean fluorescence intensity of actin filaments after NEBD

Quantification of the mean fluorescent intensity of actin filaments was done in ImageJ on two-dimensional images. To quantify the mean fluorescence intensity of actin filaments after NEBD, a region of interest of consistent size was placed next to the chromosome signal. Background was subtracted on the basis of intensity measurements in a region outside of the cell.

### Statistics and reproducibility

Average (mean), standard deviation and statistical significance were calculated in GraphPad Prism (8.4.3). Statistical tests selected for each dataset are indicated in the figure legends. All data were tested for normal distribution in GraphPad Prism (8.4.3) before selection of the statistical tests. All graphs were generated in GraphPad Prism (8.4.3), except for the graphs in Fig. 4d, which were generated in Origin 2021b, and in Fig. 4e, which were generated in MATLAB R2018b. All data from porcine oocytes are from at least two independent experiments/multiple biological replicates. The number of experimental replicates for each assay is specified in the figure legends. All replication attempts were successful. No statistical methods were used to pre-determine sample size. Due to the large size and opaque nature of porcine oocytes, cells that had nuclei positioned in the top third of the cell could not be imaged well and were hence excluded from the analysis. Cells that died during imaging were excluded from the analysis. For each independent experiment, porcine oocytes were collected from multiple ovaries/animals and subsequently pooled together. The oocytes were then randomly split into a control group and an experimental group. The investigators were not blinded to allocation during experiments and outcome assessment.

### Resource availability

#### Contact for reagent and resource sharing

Further information and requests for resources and reagents should be directed to and will be fulfilled by the corresponding author Melina Schuh (melina.schuh@mpinat.mpg.de).

### Reporting summary

Further information on research design is available in the Nature Portfolio Reporting Summary linked to this article.

## Online content

Any methods, additional references, Nature Portfolio reporting summaries, source data, extended data, supplementary information, acknowledgements, peer review information; details of author contributions and competing interests; and statements of data and code availability are available at 10.1038/s41556-022-01082-9.

## Supplementary information

Reporting Summary

Supplementary Video 1 Human oocytes cluster their chromosomes upon NEBD. Time, hours:minutes, 00:00 is NEBD. Scale bar, 10 µm. Part I: time-lapse video of chromosome clustering upon NEBD in a human oocyte. Oocyte expressing H2B-mRFP1 (magenta, chromosomes) and EGFP-MAP4 (green, microtubules). *Z*-projections, five sections every 5 µm. Oocyte also shown in Fig. 1a. Part II: time lapse of a human oocyte with chromosomes clustered around nucleolus before NEBD. Oocyte expressing H2B-mRFP1 (magenta, chromosomes) and EGFP-MAP4 (green, microtubules). *Z*-projections, five sections every 5 µm. Oocyte also shown in Extended Data Fig. 1a. Autofluorescent spots in the cytoplasm are probably lipofuscin granules.

Supplementary Video 2 Porcine oocytes cluster their chromosomes upon NEBD. Time, hours:minutes, 00:00 is NEBD. Scale bar, 10 µm. Part I: time-lapse of chromosome clustering upon NEBD in a porcine oocyte with fully dispersed chromosomes. Oocyte expressing H2B-mCherry (magenta, chromosomes) and EGFP-MAP4 (green, microtubules). *Z*-projections, 15 sections every 3 µm. Oocyte also shown in Fig. 2e. Part II: time lapse of chromosome clustering upon NEBD in a porcine oocyte with partially clustered chromosomes around nucleolus before NEBD. Oocyte expressing H2B-mCherry (magenta, chromosomes) and EGFP-MAP4 (green, microtubules). *Z*-projections, 15 sections every 3 µm. Oocyte also shown in Extended Data Fig. 2b, top. Part III: Time lapse of a porcine oocyte with chromosomes completely clustered around nucleolus before NEBD. Oocyte expressing H2B-mCherry (magenta, chromosomes) and EGFP-MAP4 (green, microtubules). *Z*-projections, 15 sections every 3 µm. Oocyte also shown in Extended Data Fig. 2b, bottom.

Supplementary Video 3 Spindle assembly in porcine oocytes is driven by RanGTP. Time, hours:minutes, 00:00 is NEBD. Scale bar, 10 µm. Oocytes expressing H2B-mCherry (magenta, chromosomes) and EGFP-MAP4 (green, microtubules). Additionally, the left oocyte expresses Ran WT-miRFP670, the right oocyte Ran T24N-miRFP670. *Z*-projections, 15 sections every 3 µm. Oocytes also shown in Fig. 2d.

Supplementary Video 4 The nuclear lamina coalesces together with chromosomes in the absence of microtubules and F-actin in porcine oocytes. Time, hours:minutes, 00:00 is NEBD. Scale bar, 10 µm. Porcine oocytes expressing H2B-mCherry (magenta, chromosomes) and EGFP-Lamin B1 (green, nuclear lamina), treated with DMSO (Part I) or nocodazole and cytochalasin D (parts II and III). *Z*-projections, six sections every 3 µm. Oocyte in part II forms the chromosome cluster. Oocyte in part III does not form the chromosome cluster. Arrowhead points to the area where nuclear lamina disappears. Oocytes also shown in Extended Data Fig. 3a, part I top panel and part II bottom panel.

Supplementary Video 5 Chromosome clustering is driven by a dynamic network of interlinked actin cables. Part I: Interlinked actin cables closely interact with the chromosomes upon NEBD. Time, hours:minutes, 00:00 is NEBD. Scale bar, 10 µm. Porcine oocyte expressing EGFP-UtrCH (grey, F-actin) and H2B-mCherry (magenta, chromosomes). White arrowheads point at areas of close contact between actin and chromosomes. Oocyte also shown in Fig. 4a. Part II: actin cables that drive chromosome clustering are highly dynamic. Time, minutes:seconds. Scale bar, 10 µm. Porcine oocyte expressing mScarlet-UtrCH (grey, F-actin) and EGFP-Lamin B1 (not shown). Oocyte also shown in Fig. 4c.

Supplementary Video 6 Actin cables interact with kinetochores during clustering. Time, minutes:seconds. Scale bar, 2 µm. Porcine oocytes expressing EGFP-UtrCH (green, F-actin), mScarlet-hCenp-C (magenta, kinetochores) and H2B-Snap-SiR647 (blue, chromosomes). White arrowheads point to kinetochore–actin interactions. Oocyte in part II was treated with nocodazole. Oocyte in part I also shown in Fig. 5c and oocyte in part II is shown in Extended Data Fig. 5c.

Supplementary Video 7 Example of kinetochore re-orientation towards the nuclear centre during the actin-dependent phase of chromosome clustering. Time, minutes:seconds. Scale bar, 10 µm. Porcine oocyte expressing mClover3-hCenpC (cyan, kinetochores) and H2B-mCherry (magenta, chromosomes). Oocyte also shown in Fig. 5d.

Supplementary Video 8 A cage of microtubule loops encapsulates the chromosome cluster. Scale bar, 5 µm. Part I: immunofluorescence airyscan image of porcine oocyte fixed 30 min after NEBD. Cyan, microtubules (α-tubulin); yellow, kinetochores (ACA); magenta, DNA (Hoechst). Image generated with Imaris 3D view. Oocyte also shown in Fig. 8a, bottom. Part II: immunofluorescence airyscan image of a human oocyte fixed 1 h after NEBD. Cyan, microtubules (α-tubulin); magenta, DNA (Hoechst); F-actin (not shown); Lamin A/C (not shown). Image generated with Imaris 3D view. Oocyte also shown in Fig. 8b, bottom.

Supplementary Video 9 Microtubule cage that assembles around the chromosomes after NEBD is stable. Scale bar, 5 µm. Part I: immunofluorescence airyscan images of porcine oocytes treated with DMSO (part I) or cytochalasin D (part II) and cold-treated before fixation. Green, microtubules (α-tubulin); magenta, kinetochores (ACA); blue, DNA (Hoechst). Images generated with Imaris 3D view.

Supplementary Tables Supplementary Table 1. Ages of human oocyte donors for data in Fig. 1 and Extended Data Fig. 1a. Supplementary Table 2. Ages of human oocyte donors for data in Figs. 4b and 8b and Extended Data Figs. 3b and 4b.

## Figures and Tables

**Extended Data Table. 9:**
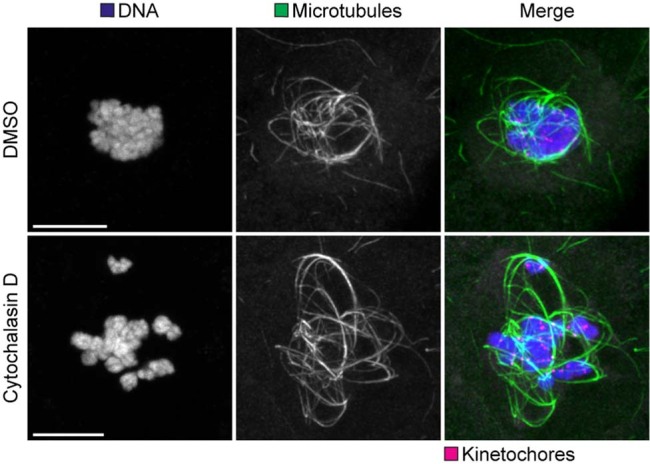
Stable microtubule loops form around the chromosomes in later stages of chromosome clustering. Immunofluorescence airyscan images of cold-resistant microtubules in porcine oocytes after NEBD treated with DMSO (top panels: Z-projections, 27 sections every 0.19 µm) or cytochalasin D (lower panels: Z-projections, 44 sections every 0.19 µm). Blue, DNA (Hoechst); green, microtubules (alpha-tubulin); magenta, kinetochores (ACA). Scale bar, 10 µm. Data from three independent experiments.

## Data Availability

Source data are provided with this paper. All other data supporting the findings of this study are available from the corresponding author on reasonable request. The primary microscopy data were not uploaded to a data repository due to their large size, but are available from the corresponding author on reasonable request.

## Source data

Source Data Fig. 1 Statistical source data.
Source Data Fig. 2 Statistical source data.
Source Data Fig. 3 Statistical source data.
Source Data Fig. 4 Statistical source data.
Source Data Fig. 5 Statistical source data.
Source Data Fig. 6 Statistical source data.
Source Data Fig. 7 Statistical source data.
Source Data Extended Data Fig./Table 1 Statistical source data.
Source Data Extended Data Fig./Table 2 Statistical source data.
Source Data Extended Data Fig./Table 3 Statistical source data.
Source Data Extended Data Fig./Table 4 Statistical source data.
Source Data Extended Data Fig./Table 5 Statistical source data.
Source Data Extended Data Fig./Table 6 Statistical source data.
Source Data Extended Data Fig./Table 7 Statistical source data.
Source Data Extended Data Fig./Table 8 Statistical source data.
Source Data Extended Data Fig./Table 8 Unprocessed western blot.

## Code availability

Scripts and plugins are available at: https://gitlab.gwdg.de/schuh-meiosis/Actin-driven-chromosome-clustering-facilitates-fast-and-complete-chromosome-capture-in-mammalian-oocytes.
