# Publisher Correction: Actin-driven chromosome clustering facilitates fast and complete chromosome capture in mammalian oocytes

**DOI:** 10.1038/s41556-023-01156-2

**Published:** 2023-05-04

**Authors:** Katarina Harasimov, Julia Uraji, Eike Urs Mönnich, Zuzana Holubcová, Kay Elder, Martyn Blayney, Melina Schuh

**Affiliations:** 1 Max Planck Institute for Multidisciplinary Sciences, Göttingen, Germany; 2 Bourn Hall Clinic, Cambridge, UK; 3 Medical Research Council Laboratory of Molecular Biology, Cambridge, UK; 4 Cluster of Excellence ‘Multiscale Bioimaging: from Molecular Machines to Networks of Excitable Cells’ (MBExC), University of Göttingen, Göttingen, Germany

**Keywords:** Cytoskeleton, Developmental biology, Chromosomes

Correction to: *Nature Cell Biology* 10.1038/s41556-022-01082-9. Published online 2 February 2023.

In the version of this article initially published, Supplementary Videos 1–8 were linked out of order, which caused Supplementary Video 1 to be inaccessible. The Supplementary Videos are now appropriately linked in the HTML version of the article.

