# Supplementary material for: Actin-driven chromosome clustering facilitates fast and complete chromosome capture in mammalian oocytes
Source: Nat Cell Biol. 2023 Feb 2;25(3):439–52. doi: 10.1038/s41556-022-01082-9 (PMC10014578; doi:10.1038/s41556-022-01082-9)
Supplement: Supplementary file 1 — Reporting Summary [file 41556_2022_1082_MOESM1_ESM.pdf]

nature portfolio

Corresponding author(s): Melina Schuh

Last updated by author(s): Dec 1, 2022

Reporting Summary

Nature Portfolio wishes to improve the reproducibility of the work that we publish. This form provides structure for consistency and transparency in reporting. For further information on Nature Portfolio policies, see our Editorial Policies and the Editorial Policy Checklist.

Statistics

For all statistical analyses, confirm that the following items are present in the figure legend, table legend, main text, or Methods section.

n/a	Confirmed	
☐	☒	The exact sample size (*n*) for each experimental group/condition, given as a discrete number and unit of measurement
☐	☒	A statement on whether measurements were taken from distinct samples or whether the same sample was measured repeatedly
☐	☒	The statistical test(s) used AND whether they are one- or two-sided<br>*Only common tests should be described solely by name; describe more complex techniques in the Methods section.*
☒	☐	A description of all covariates tested
☐	☒	A description of any assumptions or corrections, such as tests of normality and adjustment for multiple comparisons
☐	☒	A full description of the statistical parameters including central tendency (e.g. means) or other basic estimates (e.g. regression coefficient) AND variation (e.g. standard deviation) or associated estimates of uncertainty (e.g. confidence intervals)
☐	☒	For null hypothesis testing, the test statistic (e.g. *F*, *t*, *r*) with confidence intervals, effect sizes, degrees of freedom and *P* value noted<br>*Give P values as exact values whenever suitable.*
☒	☐	For Bayesian analysis, information on the choice of priors and Markov chain Monte Carlo settings
☒	☐	For hierarchical and complex designs, identification of the appropriate level for tests and full reporting of outcomes
☒	☐	Estimates of effect sizes (e.g. Cohen's *d*, Pearson's *r*), indicating how they were calculated

Our web collection on statistics for biologists contains articles on many of the points above.

Software and code

Policy information about availability of computer code

Data collection	Zen Blue 2.3, Zen Blue 3.0 and Zen Black 2.1. Automatic 3D tracking was implemented for time-lapse imaging with a temporal resolution between 1.5 and 10 min using AutofocusScreen (PMID: 15516224) or MyPiC (PMID: 29844523) on the LSM880, or by using a customized sample tracking solution provided by ZEISS Microscopy on LSM800 and LSM900 microscopes.
Data analysis	Statistical analysis was performed in GraphPad Prism (8.4.3). All graphs were generated in GraphPad Prism (8.4.3), except the graphs in Fig. 4d which was generated in Origin 2021b, and in Fig. 4e which was generated in MATLAB R2018b. Images were analysed using Imaris 8.4.1 or Imaris 9.3.1 . In-house developed scripts for data analysis within Imaris and Matlab are available at: https://gitlab.gwdg.de/schuh-meiosis/Actin-driven-chromosome-clustering-facilitates-fast-and-complete-chromosome-capture-in-mammalian-oocytes

For manuscripts utilizing custom algorithms or software that are central to the research but not yet described in published literature, software must be made available to editors and reviewers. We strongly encourage code deposition in a community repository (e.g. GitHub). See the Nature Portfolio guidelines for submitting code & software for further information.

Data

Policy information about availability of data

All manuscripts must include a data availability statement. This statement should provide the following information, where applicable:

- Accession codes, unique identifiers, or web links for publicly available datasets
- A description of any restrictions on data availability
- For clinical datasets or third party data, please ensure that the statement adheres to our policy

All relevant and raw data supporting the finding of this study are available from the corresponding author on reasonable request. The primary microscopy data were not uploaded to a data repository due to their large size, but are available from the corresponding author on reasonable request.

Human research participants

Policy information about studies involving human research participants and Sex and Gender in Research.

Reporting on sex and gender	All donors in this study were of female sex.
Population characteristics	All donations were provided anonymously. The age of all oocyte donors is provided in the supplementary information file in the subsection titled "Human oocyte donor ages".
Recruitment	All human oocytes obtained for this study were sourced from patients undergoing fertility treatments at Bourn Hall Clinic (Bourn, Cambridgeshire, UK) between 12.01.2017 and 24.07.2019 after having obtained fully informed consent. All donations were anonymous. Patients were not monetarily compensated for their donation. Donors were recruited by the nursing staff at the Bourn Hall Clinic. Authors of this paper did not directly take part in the recruitment process. All donors underwent ovarian stimulation for intracytoplasmic sperm injection (ICSI). Only oocytes that were immature and hence unsuitable for ICSI were donated to the study.
Ethics oversight	The use of immature unfertilized human oocytes in this study has been approved by the United Kingdom's National Research Ethics Service under the REC reference 11/EE/0346 (IRAS Project ID 84952).

Note that full information on the approval of the study protocol must also be provided in the manuscript.

Field-specific reporting

Please select the one below that is the best fit for your research. If you are not sure, read the appropriate sections before making your selection.

☒ Life sciences ☐ Behavioural & social sciences ☐ Ecological, evolutionary & environmental sciences

For a reference copy of the document with all sections, see nature.com/documents/nr-reporting-summary-flat.pdf

Life sciences study design

All studies must disclose on these points even when the disclosure is negative.

Sample size	No statistical methods were used to predetermine sample size. In retrospective, achieved sample sizes were determined to be adequate based on the magnitude and consistency of measurable differences between groups. Most importantly, sample size per experiment was dictated by the number of oocytes that could be processed (number of ovaries available from the slaughterhouse, microinjection, and live imaging) within a reasonable time by the researcher without affecting oocyte quality. Following parameters further dictated final sample size per experiment, which varied on different experimental days:<br>1. Number of antral follicles per porcine ovary<br>2. Number of female animals processed in the slaughterhouse<br>3. Survival rate of oocytes upon injection<br>4. Nuclear envelope breakdown rate.
Data exclusions	Due to the large size and opaque nature of porcine oocytes, cells that had nucleus positioned in the top third of the cell (away from the coverslip) could not be imaged well and were hence excluded from the analysis. Cells that died during imaging were excluded from the analysis.
Replication	All data from porcine oocytes are from at least two independent experiments / multiple biological replicates. All attempts at replication were successful.
Randomization	For each independent experiment, porcine oocytes were collected from multiple ovaries/animals, and subsequently pooled. They were then randomly split into a control group, and an experimental group (where e.g. a protein was inhibited).
Blinding	Investigators were not blinded to allocation during experiments and outcome assessment, as each experiment was performed by one researcher alone. Thus, blinding during group allocation was not possible to ensure samples received the right treatment/manipulation during

experiment. Blinding was also not possible during data analysis, as data analysis in most cases was performed by the same researcher that conducted the experiment.

Reporting for specific materials, systems and methods

We require information from authors about some types of materials, experimental systems and methods used in many studies. Here, indicate whether each material, system or method listed is relevant to your study. If you are not sure if a list item applies to your research, read the appropriate section before selecting a response.

Materials & experimental systems

n/a	Involved in the study	
☐	☒	Antibodies
☒	☐	Eukaryotic cell lines
☒	☐	Palaeontology and archaeology
☐	☒	Animals and other organisms
☒	☐	Clinical data
☒	☐	Dual use research of concern

Methods

n/a	Involved in the study	
☒	☐	ChIP-seq
☒	☐	Flow cytometry
☒	☐	MRI-based neuroimaging

Antibodies

Antibodies used

Primary antibodies used were rat anti-α-tubulin (clone: YOL1/34, #MCA78G, Bio-Rad), rabbit anti-Fmn2 (#HPA050649, Atlas antibodies), mouse anti-Lamin A/C antibody (clone: 4C11, #SAB4200236, Sigma-Aldrich), and human anti-centromere antibody (ACA) (#15-234, Antibodies Incorporated). Secondary antibodies used were Goat anti-Rat IgG (H+L) Cross-Adsorbed Secondary Antibody, Alexa Fluor 647 (#A-21247, ThermoFisher), Goat anti-Human IgG (H+L) Cross-Adsorbed Secondary Antibody, Alexa Fluor 488 (#A-11013, ThermoFisher), Goat anti-Human IgG (H+L) Cross-Adsorbed Secondary Antibody, Alexa Fluor 568 (#A-21090, ThermoFisher), Donkey anti-Mouse IgG (H+L) Highly Cross-Adsorbed Secondary Antibody, Alexa Fluor 568 (#A10037, ThermoFisher), Goat anti-Mouse IgG (H+L) Cross-Adsorbed Secondary Antibody, Alexa Fluor 546 (A-11003, ThermoFisher), Goat anti-Rabbit IgG (H+L) Cross-Adsorbed Secondary Antibody, Alexa Fluor 568 (A-11011, ThermoFisher), Donkey anti-Rabbit IgG (H+L) Highly Cross-Adsorbed Secondary Antibody, Alexa Fluor 568 (#A-10042, ThermoFisher), Goat anti-Rabbit IgG (H+L) Cross-Adsorbed Secondary Antibody, Alexa Fluor 546 (#A-11010, ThermoFisher), Goat anti-Rabbit IgG (H+L) Cross-Adsorbed Secondary Antibody, HRP (#31462, , ThermoFisher).

Validation

The rabbit anti-Fmn2 (#HPA050649, Atlas antibodies) was validated with immunofluorescence in human cells on the Human Protein Atlas (https://www.proteinatlas.org/ENSG00000155816-FMN2/cell). Localized to the plasma membrane and actin filaments. In this paper, we validated this antibody in porcine oocytes on a Western Blot, and with immunofluorescence. The antibody gave a single band on a Western Blot from porcine oocytes at approximately 180 kDa. In immunofluorescence, the antibody localized to actin filaments in porcine oocytes.
The rat anti-α-tubulin antibody (clone: YOL1/34, #MCA78G, Bio-Rad) is routinely tested in ELISA on Tubulin by the manufacturer (statement from the manufacturers' webpage). This antibody was tested by the manufacturer for specificity on human α-tubulin. Based on high sequence homology, this antibody is expected to bind to α-tubulin from in all mammals (statement from the manufacturers' webpage). This rat anti-α-tubulin antibody was previously used on porcine oocytes (PMID: 35143306 and 31679939), and showed a staining pattern that matched the observations from live cell imaging of microtubules. In this paper we also validate this antibody with immunofluorescence in porcine oocytes, where it localized to the microtubule spindle.
The mouse anti-Lamin A/C antibody (clone: 4C11, #SAB4200236, Sigma-Aldrich) was validated by the manufacturer on a Western Blot, and with immunofluorescence. Localized to the nuclear envelope. The antibody is predicted to work with human, mouse, monkey, bovine, canine, rat, hamster samples. In this paper we validate this antibody with immunofluorescence in porcine oocytes, where it localized to the nuclear envelope.
The human anti-centromere antibody (ACA) (#15-234, Antibodies Incorporated) is a commonly used antibody serum derived from human CREST patient serum. The antibody is tested for specificity to hamster, human, mouse and rat cells by the manufacturer. In this paper we validate this antibody with immunofluorescence in porcine oocytes, where it localized to the centromere region on the chromosomes, as expected from previously observed staining patterns in other species validated by the manufacturer.

Animals and other research organisms

Policy information about studies involving animals; ARRIVE guidelines recommended for reporting animal research, and Sex and Gender in Research

Laboratory animals

No laboratory animals were used in this study.

Wild animals

No wild animals were used in this study.

Reporting on sex

This study investigates the development of female reproductive cells called oocytes. We only used ovaries from female pigs or immature oocytes from women, but no materials from males. Porcine ovaries used in this study were collected by the employees at a local abattoir. Immature human oocytes were donated by women undergoing fertility treatment.

Field-collected samples	No field-collected samples were used in this study.
Ethics oversight	The porcine ovaries used in this study were obtained from a local abattoir as a waste product of the slaughtering process, and as such do not require ethics approval for usage in Germany where the study was conducted. All human oocytes obtained for this study were sourced from patients undergoing fertility treatment at Bourn Hall Clinic (Bourn, Cambridgeshire, UK) between 12.01.2017 and 24.07.2019 after having obtained informed consent. The use of immature unfertilized human oocytes has been approved by the UK's National Research Ethics Service under the REC reference 11/EE/0346 (IRAS Project ID 84952).

Note that full information on the approval of the study protocol must also be provided in the manuscript.
