# Supplementary figures and images for: Actin-driven chromosome clustering facilitates fast and complete chromosome capture in mammalian oocytes
Source: Nat Cell Biol. 2023 Feb 2;25(3):439–52. doi: 10.1038/s41556-022-01082-9 (PMC10014578; doi:10.1038/s41556-022-01082-9)

30 oocytes, Atlas antibody  
anti-FMN<sub>2</sub>

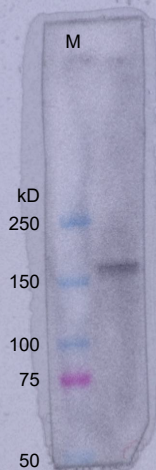

Supplement: Source Data Extended Data Fig./Table 8 — Unprocessed western blot. [file 41556_2022_1082_MOESM27_ESM.pdf]
